# Long non-coding RNA *CASC19* is associated with the progression and prognosis of advanced gastric cancer

**DOI:** 10.18632/aging.102190

**Published:** 2019-08-15

**Authors:** Wen-Jie Wang, Chang-An Guo, Rui Li, Zi-Peng Xu, Jian-Ping Yu, Yan Ye, Jun Zhao, Jing Wang, Wen-An Wang, An Zhang, Hong-Tao Li, Chen Wang, Hong-Bin Liu

**Affiliations:** 1 Second Clinical Medical College, Lanzhou University, Lanzhou 730030, Gansu, P.R. China; 2 Department of General Surgery, Lanzhou University Second Hospital, Lanzhou 730030, Gansu, P.R. China; 3 Department of General Surgery, The 940th Hospital of Joint Logistics Support Force of Chinese People’s Liberation Army, Lanzhou 730050, Gansu, P.R. China; 4 Key Laboratory of Stem Cells and Gene Drugs of Gansu Province, Lanzhou 730050, Gansu, China; 5 Department of Emergency, Lanzhou University Second Hospital, Lanzhou 730030, Gansu, P.R. China; 6 Clinical Medical College, Gansu University of Chinese Medicine, Lanzhou 730000, Gansu, P.R. China

**Keywords:** gastric cancer, cancer susceptibility 19, progression, prognosis, weighted gene co-expression network analysis

## Abstract

Evidence indicates that aberrantly expressed long non-coding RNAs (lncRNAs) are involved in the development and progression of advanced gastric cancer (AGC). Using RNA sequencing data and clinical information obtained from The Cancer Gene Atlas, we combined differential lncRNA expression profiling and weighted gene co-expression network analysis to identify key lncRNAs associated with AGC progression and prognosis. Cancer susceptibility 19 (*CASC19*) was the top hub lncRNA among the lncRNAs included in the gene module most significantly correlated with AGC’s pathological variables. *CASC19* was upregulated in AGC clinical samples and was significantly associated with higher pathologic TNM stage, pathologic T stage, lymph node metastasis, and poor overall survival. Multivariable Cox analysis confirmed that *CASC19* overexpression is an independent prognostic factor for overall survival. Furthermore, quantitative real-time PCR assay confirmed that *CASC19* expression in four human gastric cancer cells (AGS, BGC-823, MGC-803, and HGC-27) was significantly upregulated compared with human normal gastric mucosal epithelial cell line (GES-1). Functionally, *CASC19* knockdown inhibited GC cell proliferation and migration in vitro. These findings suggest that *CASC19* may be a novel prognostic biomarker and a potential therapeutic target for AGC.

## INTRODUCTION

Gastric cancer (GC) is one of the most common malignant tumors of the digestive tract worldwide, and its morbidity and mortality are ranked 4^th^ and 2^nd^, respectively, among all malignant tumors [1, 2]. Approximately two-thirds of patients present with advanced GC (AGC) at initial diagnosis, and their 5-year survival rate is only 20%–30%. Therefore, screening novel biomarkers to identify reliable therapeutic targets has become an urgent issue for the prevention and treatment of GC.

Long non-coding RNAs (lncRNAs) are a class of RNA molecules greater than 200 nucleotides in length that do not encode proteins [3, 4]. LncRNAs are abundantly present in the human transcriptome and may exert oncogenic or tumor suppressor effects [5–7] by affecting expression of target genes at the epigenetic, transcriptional, and post-transcriptional levels, and by participating in such processes as chromatin modification, genomic imprinting, and intranuclear transport [8–10]. Moreover, several studies have reported an association between aberrantly expressed lncRNAs and the development and progression of AGC [11–14]. However, the specific molecular mechanisms remain unclear.

High-throughput RNA sequencing is widely used to investigate lncRNA expression and regulation in cancer and has provided new insights into oncogenic mechanisms and potential therapeutic targets [15]. However, most studies have mainly addressed individual lncRNAs, rarely focusing on genome-wide correlations between differentially expressed lncRNAs and clinical traits [16–18]. In recent years, weighted gene co-expression network analysis (WGCNA) has been used to study the association between gene sets and clinical features to identify novel prognostic biomarkers and potential therapeutic targets [19, 20]. WGCNA quantitatively assesses the degree of association between lncRNAs and captures the complex relationship between lncRNAs and phenotypes, thereby providing an effective way to explore the mechanisms behind certain clinical features.

We applied an innovative genomic analysis method that combined differential lncRNAs expression analysis with WGCNA, and evaluated RNA sequencing data from The Cancer Genome Atlas (TCGA) to identify key lncRNAs associated with the development and progression of AGC. In addition, gene set enrichment analysis (GSEA) was conducted to explore signaling pathways related to key lncRNAs. Our results demonstrate the relevance of the hub lncRNA *CASC19* as predictor of tumor stage and overall survival, and suggest *CASC19* is a novel biomarker of AGC and a potential therapeutic target.

## RESULTS

### Patient characteristics

**Figure 1:**
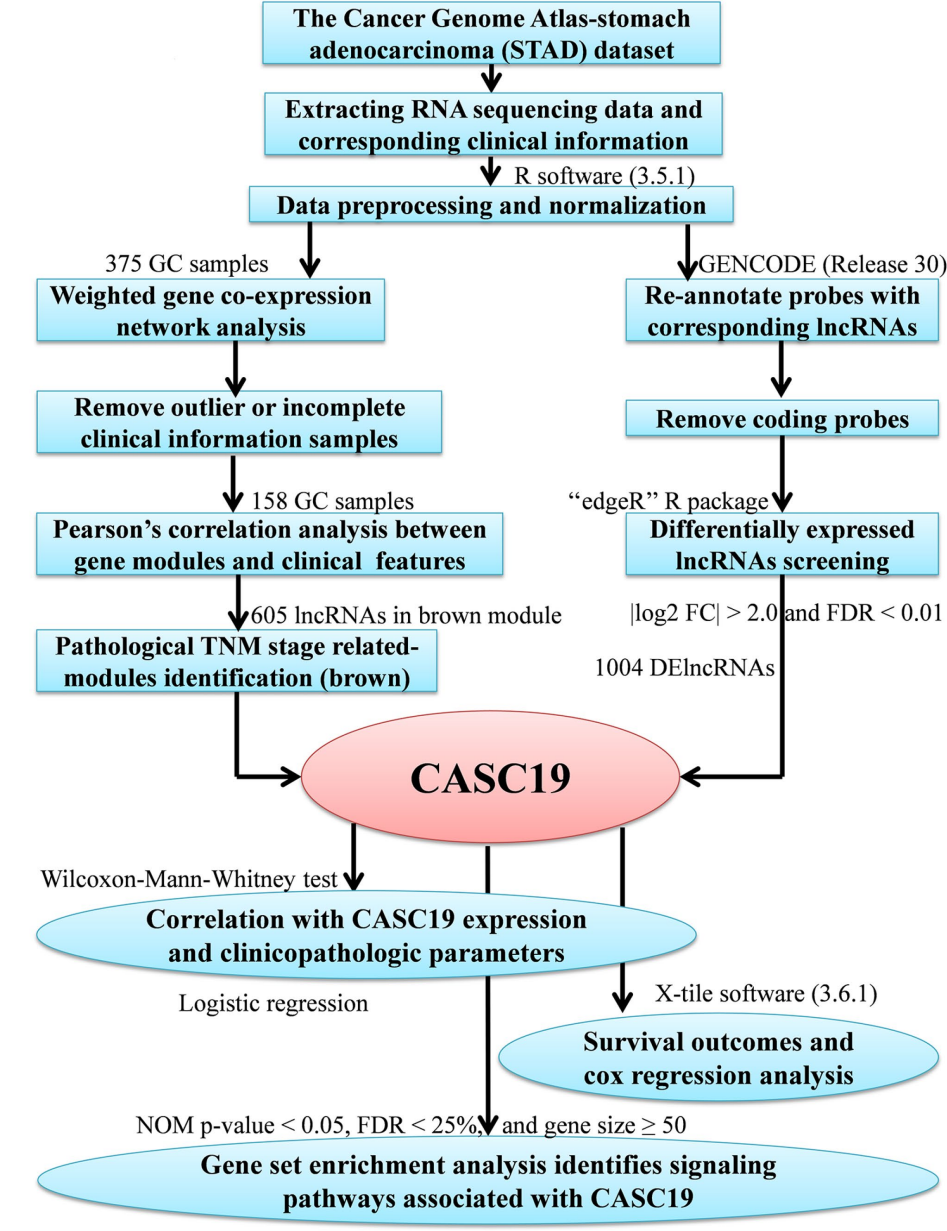
**Study analysis flowchart.**

The study’s workflow is presented in Figure 1. Three hundred and fifty-one AGC patients with complete clinical information were eventually included in the study. The median age at which the patients were diagnosed with gastric cancer was 67 years old, and median follow-up time was 13.1 months (range 0–124 months). Detailed demographics and clinicopathologic parameters of AGC patients are provided in Table 1.

**Table 1:** Demographics and clinicopathologic characteristics of AGC patients.

**Variables**	**Total (N)**	**%**
Age (years)		
<65 years	149	42.5
≥65 years	202	57.5
Gender		
Male	224	63.8
Female	127	36.2
Histologic type		
Well	9	2.6
Moderate	117	33.3
Poor	216	61.5
Pathologic TNM stage		
I	34	9.7
II	109	31.1
III	149	42.5
IV	37	10.5
Pathologic T stage		
T2	75	21.4
T3	167	47.6
T4	100	28.5
Metastatic lymph nodes		
Negative	95	27.1
Positive	238	67.8
Distant metastasis		
Negative	309	88.0
Positive	25	7.1
Status		
Alive	212	60.4
Dead	139	39.6

### Identification of differentially expressed lncRNAs

A total of 1004 differentially expressed lncRNAs (DElncRNAs) were screened out between GC tissues and non-tumor tissues using “edgeR” R package with |log_2_ (fold change [FC])| > 2.0 and false discovery rate (FDR) < 0.01 as thresholds. Among those, 790 (78.7%) were upregulated and 214 (21.3%) were downregulated in GC tissues. All these DElncRNAs were chosen for subsequent analysis (Supplementary Table 1).

### Co-expression network construction and identification of significant modules

**Figure 2:**
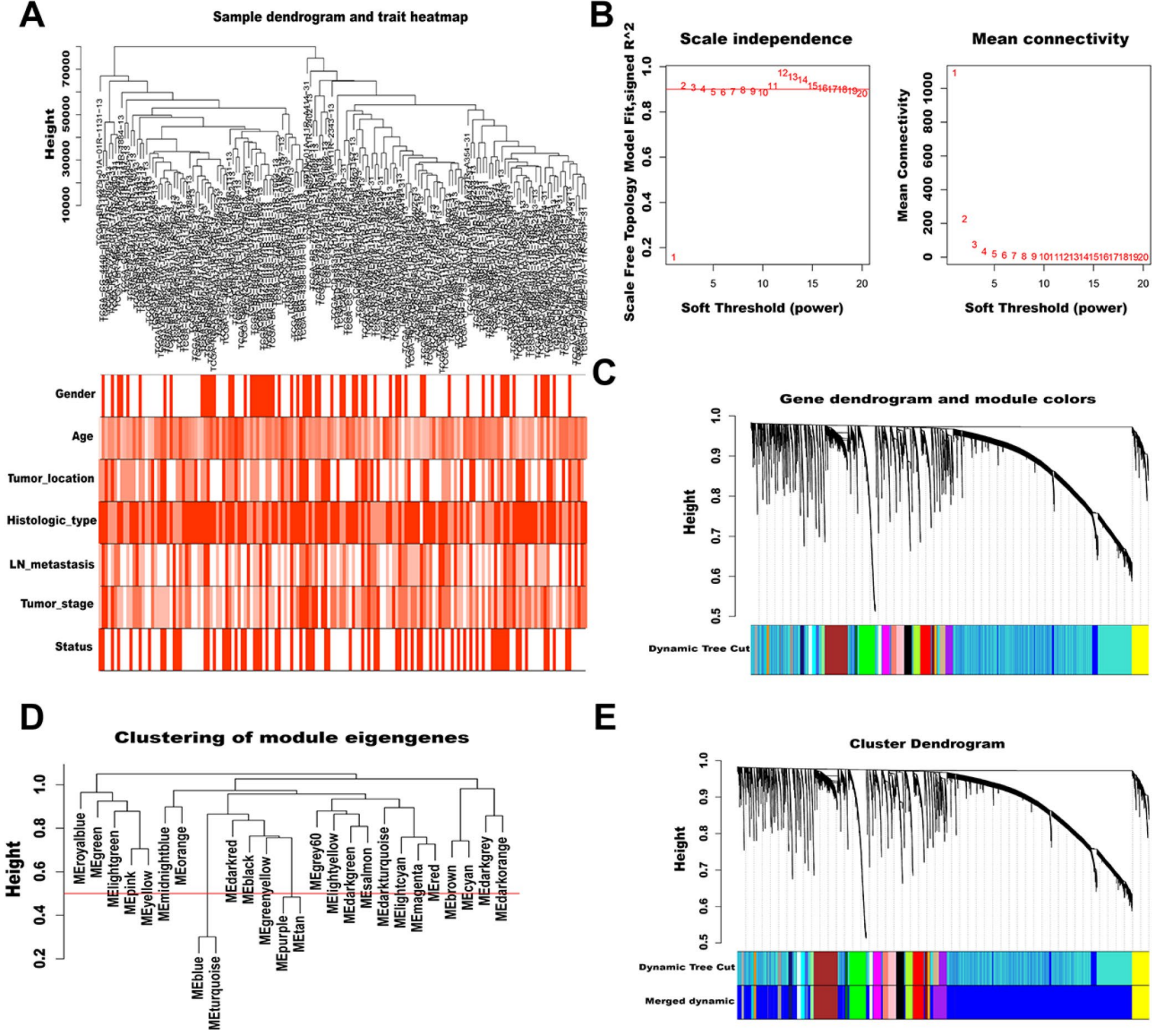
**WGCNA of lncRNAs in AGC.** (**A**) Sample dendrogram and trait heatmap (outliers and samples with incomplete clinical information were removed). Color depth is proportional to the strength of the correlation with clinical traits in each sample, with red and white representing highest and lowest correlation, respectively. (**B**) Soft-thresholding power analysis of scale independence and mean connectivity. The left graph shows the correlation coefficients that correspond to different soft-thresholding powers. The higher the coefficient, the more the network conforms to the distribution of scale-free networks. The right graph displays the mean coefficient of contiguous genes in the gene network corresponding to different soft-thresholding powers, which reflects the average connection level of the network. (**C**) The dynamic Tree Cut method classifies gene clustering trees. Different colors represent different gene modules, and gray indicates genes that do not belong to any known module. (**D**) Cluster dendrogram of module eigengenes. The value corresponding to the red line in the figure indicates the merge threshold. (**E**) Clustering dendrogram of genes by hierarchical clustering based on the dissimilarity TOM. Dynamic tree cut corresponds to the originally obtained module, and merged dynamic corresponds to the merged module finally obtained.

To characterize lncRNAs profiles in AGC, a co-expression network was constructed by WGCNA. After removing outlier samples and those with incomplete clinical information, 158 samples were used to construct an adjacency matrix (Figure 2A). We selected β = 2 as the soft thresholding power to ensure a correlation coefficient close to 0.9 (Figure 2B). Then, a total of 26 different color-coded co-expression modules were identified by the dynamic Tree Cut method, and minimum lncRNA number in each cluster tree was set to 60 (Figure 2C). After combining highly similar modules (MEDissThres = 0.5, Figure 2D), 24 modules were generated, each containing 60 to 6391 lncRNAs (Figure 2E).

**Figure 3:**
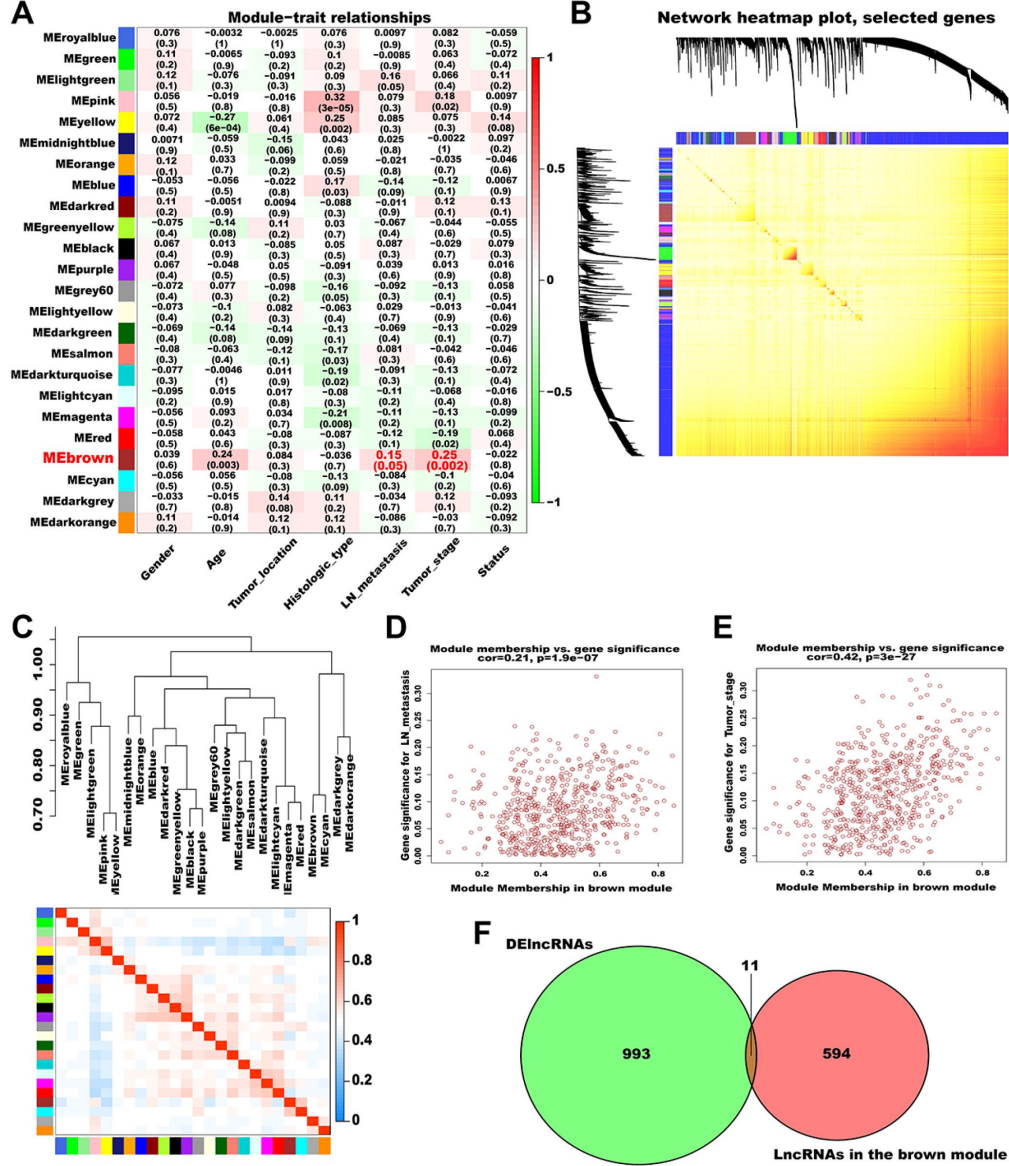
**Identification of significant modules associated with clinical traits.** (**A**) Relationships between module eigengenes and clinical traits of AGC. Each row in the figure corresponds to a module eigengene, and each column corresponds to a clinical trait. The correlation coefficient in each grid represents the correlation between the gene module and the clinical traits; red indicates positive correlation and green represents negative correlation. (**B**) TOM depicting the correlation of pairs of genes within each module. The heat map depicts the TOM from 1000 randomly selected genes from a weighted co-expression network. In the heat map, each row and column correspond to a gene; light colors indicate low topological overlap, and progressively darker yellow and red represent higher topological overlap. (**C**) Dendrogram heatmap of the association between modules and clinical traits. The dendrogram above shows the modules generated in the cluster analysis. Branches of the dendrogram combine positively correlated eigengenes. The heat map below shows the adjacencies in the eigengene network. Each row and column in the heat map corresponds to a module eigengene. Red indicates a positive correlation with high adjacency and blue indicates a negative correlation with low adjacency. The red square along the diagonal is the meta-module. (**D**) Scatter plot of MM versus GS for LN metastasis (cor = 0.21, P = 1.9e-07) in the brown module. (**E**) Scatter plot of MM versus GS for pathological TNM stage (cor = 0.42, P = 3e-27) in the brown module. (**F**) Venn plot of DElncRNAs and the lncRNAs in the brown module. Green represents the DElncRNAs and red represents the lncRNAs in the brown module.

Subsequently, we analyzed the association between the generated modules and AGC clinical features (Figure 3A). The brown module (contained 605 lncRNAs) was significantly correlated with lymph node (LN) metastasis and pathological TNM stage and was selected as a significant module for further analyses. A topological overlap matrix (TOM) plot showed that each module in the network was independent of each other, further indicating that gene expression in each module was also relatively independent (Figure 3B). Therefore, we explored the co-expression similarity of all modules using eigengenes and detected three main sub-clusters (Figure 3C). Besides, we generated scatter plots of module membership (MM) versus gene significance (GS) for LN metastasis and pathological TNM stage in the brown module, and a high correlation was obtained in all cases (LN metastasis: cor = 0.21, *P* = 1.9e-07; pathological TNM stage: cor = 0.42, *P* = 3e-27; Figure 3D and 3E).

### Hub lncRNAs screening

To screen hub lncRNAs, we matched the DElncRNAs with the lncRNAs in the brown module using the “VennDiagram” R package. In total, 11 overlapping lncRNAs (*AC108463.3*, *AL135924.2*, *AC008114.1*, *AL512413.1*, *MYB-AS1*, *AC012467.1*, *AP005233.2*, *KRT7-AS*, *CASC19*, *AC010998.1*, and *AC012668.3*) were identified and considered as hub lncRNAs (Figure 3F, Supplementary Table 2). Among these, *CASC19* had the highest MM and GS and was thus selected for deeper analysis and validation.

**Table 2:** CASC19 overexpression associated with clinical pathological characteristics of GC patients.

**Variables**	**Total (N)**	**OR (95% CI)**	**P**
Age (years)			
<65 years	149	Ref.	
≥65 years	202	1.104 (0.684-1.773)	0.684
Gender			
Male	224	Ref.	
Female	127	1.024 (0.629-1.683)	0.926
Histology			
Well	9	Ref.	
Moderate or poor	333	0.338 (0.018-1.877)	0.309
Pathologic TNM stage			
I-II	143	Ref.	
III-IV	186	1.942 (1.251-3.032)	0.003
Pathologic T stage			
T2	76	Ref.	
T3-T4	267	1.813 (1.045-3.110)	0.032
Metastatic lymph nodes			
Negative	95	Ref.	
Positive	238	2.706 (1.653-4.503)	<0.001
Distant metastasis			
Negative	309	Ref.	
Positive	25	2.090 (0.769-7.319)	0.188

Bold values indicate P < 0.05.

### Correlation between *CASC19* expression and clinicopathologic parameters

**Figure 4:**
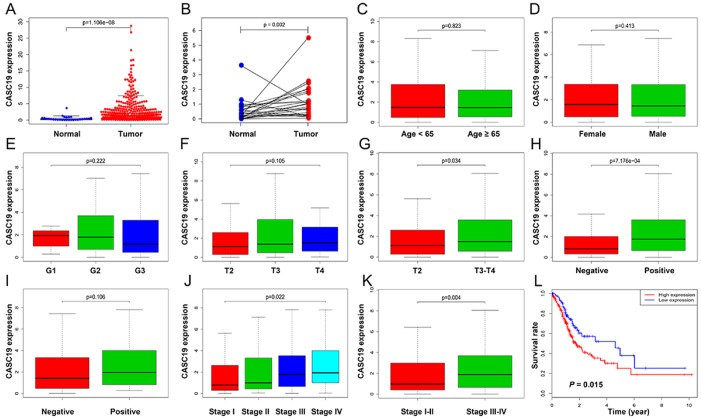
**Correlation between *CASC19* expression and clinicopathologic parameters.** (**A**) *CASC19* expression comparison between AGC tissues and non-tumor tissues. (**B**) *CASC19* expression comparison between AGC tissues and paired non-tumor tissues. (**C**) *CASC19* expression comparison between different age groups. (**D**) *CASC19* expression comparison between genders. (**E**) *CASC19* expression comparison based on tumor histology. (**F**) and (**G**) *CASC19* expression comparison between different pathologic T stages. (**H**) *CASC19* expression based on metastatic LN status. (**I**) *CASC19* expression based on distant metastasis status. (**J**) and (**K**) *CASC19* expression comparison between different pathologic TNM stages. (**L**) Kaplan–Meier survival curves. AGC patients with high *CASC19* expression (≥ 0.57) had significantly worse prognosis than those with low *CASC19* expression (< 0.57) for overall survival.

The upregulation of *CASC19* in AGC samples (Figure 4A and 4B) was significantly associated with pathologic T stage (*P* = 0.034), pathologic TNM stage (*P* = 0.022), and LN metastasis (*P* < 0.001), but not with other clinicopathologic parameters (Figure 4C–4K). The optimal cutoff value for *CASC19* was confirmed as 0.57 by using X-tile software. Next, AGC patients were divided into high (n = 256 cases) and low (n = 95 cases) *CASC19* expression groups based on the optimal cutoff value. Univariate logistic regression using R software indicated that *CASC19* overexpression significantly correlated with higher pathologic TNM stage (I-II vs. III-IV; Odds ratio [OR] = 1.942, 95% confidence interval [CI] = 1.251-3.032, *P* = 0.003), higher pathologic T stage (T2 vs. T3-T4; OR = 1.813, 95% CI = 1.045-3.110, *P* = 0.032), and LN metastasis (negative vs. positive; OR = 2.706, 95% CI = 1.653-4.503, *P* < 0.001) (Table 2). These results revealed that AGC patients with *CASC19* overexpression are more susceptible to carcinogenesis and progression.

### *CASC19* overexpression predicts poor prognosis in AGC

AGC patients with high *CASC19* expression (≥ 0.57) had significantly worse prognosis than those with low *CASC19* expression (< 0.57) for overall survival (Figure 4L, *P* = 0.015). Univariate Cox analysis showed that *CASC19* expression was associated with shorter overall survival (low vs. high; Hazard Ratio [HR] = 1.637, 95% CI = 1.096-2.447, *P* = 0.016), and so were age (*P* = 0.016), pathologic TNM stage (*P* = 0.005), and LN metastasis (*P* = 0.036). Multivariable Cox analysis confirmed that *CASC19* overexpression was an independent prognostic factor for overall survival (low vs. high; HR = 1.524, 95% CI = 1.003-2.316, *P* = 0.049; Table 3).

**Table 3:** Univariate and multivariate analysis of the correlation of CASC19 expression with OS among GC patients.

**Variables**	**Univariate analysis**			**Multivariate analysis**		
	**HR**	**95% CI**	**P**	**HR**	**95% CI**	**P**
Age (years)						
<65 years	Ref.			Ref.		
≥65 years	1.542	1.085-2.192	**0.016**	1.585	1.096-2.291	**0.014**
Gender						
Male	Ref.					
Female	0.857	0.599-1.226	0.399			
Histology						
Well	Ref.					
Moderate or poor	1.931	0.477-7.814	0.356			
Pathologic TNM stage						
I-II	Ref.			Ref.		
III-IV	1.699	1.175-2.456	**0.005**	1.501	0.902-2.499	0.118
Pathologic T stage						
T2	Ref.					
T3-T4	1.470	0.955-2.262	0.080			
Metastatic lymph nodes						
Negative	Ref.					
Positive	1.573	1.031-2.400	**0.036**	1.123	0.623-2.024	0.700
Distant metastasis						
Negative	Ref.					
Positive	1.760	0.945-3.279	0.074			
CASC19 expression						
Low	Ref.			Ref.		
High	1.637	1.096-2.447	**0.016**	1.524	1.003-2.316	**0.049**

Bold values indicate P < 0.05.

### GSEA identifies signaling pathways associated with *CASC19*

**Figure 5:**
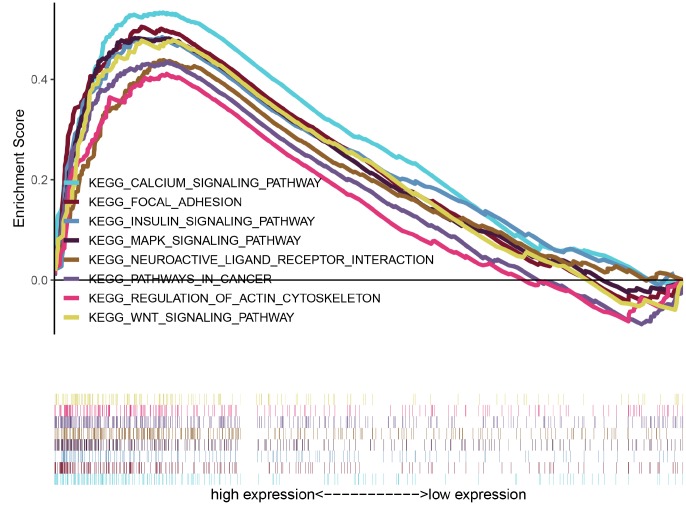
**GSEA identifies eight representative pathways enriched in AGC samples with high *CASC19* expression.**

To explore potential signaling pathways relating *CASC19* to AGC, GSEA was empolyed to identify Kyoto Encyclopedia of Genes and Genomes (KEGG) pathways enriched in AGC samples with high *CASC19* expression. Results highlighted 30 enriched gene sets with Nominal (NOM) *p* < 0.05, FDR < 25%, and gene size ≥ 50. Interestingly, most of the gene sets are concentrated on ‘cancer-related’ and ‘classical signaling’ pathways. The top eight representative pathways were “pathways in cancer”, “neuroactive ligand receptor interaction”, “MAPK signaling pathway”, “regulation of actin cytoskeleton”, “focal adhesion”, “calcium signaling pathway”, “wnt signaling pathway” and “insulin signaling pathway” (Figure 5 and Supplementary Table 3).

### *CASC19* knockdown inhibits GC cell proliferation and metastasis

**Figure 6:**
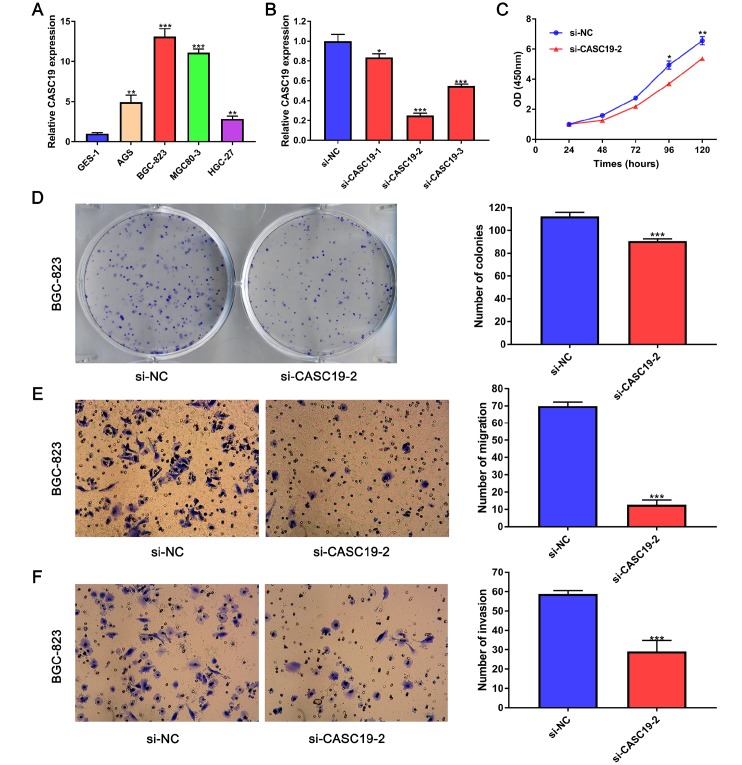
***CASC19* knockdown inhibits GC cell proliferation and metastasis.** (**A**) qRT-PCR analysis of *CASC19* expression in four GC cell lines (AGS, BGC-823, MGC-803, and HGC-27) and a normal gastric mucosal epithelial cell line (GES-1). (**B**) qRT-PCR showing successful *CASC19* knockdown in BGC-823 cells using si-*CASC19*-2. (**C**) *CASC19* knockdown inhibits proliferation in BGC-823 cells. (**D**) Colony formation is increased after *CASC19* knockdown. (**E**) and (**F**) *CASC19* silencing decreases cell migration in Transwell assays. All data are presented as mean ± standard deviation of three independent experiments. ^*^P < 0.05;^**^P < 0.01; ^***^ *P* < 0.001.

To further investigate the role of *CASC19* in GC progression, we firstly determined *CASC19* expression in four human GC cell lines (AGS, BGC-823, MGC-803, and HGC-27) and in a normal gastric mucosal epithelial cell line (GES-1) by quantitative real-time PCR (qRT-PCR). *CASC19* expression was upregulated in all four GC cell lines, and especially in BGC-823 cells, compared to GES-1 cells (*P* < 0.001; Figure 6A). Thus, we selected the BGC-823 cell line to further explore the pathogenic mechanism of *CASC19* in GC. To this end, *CASC19* expression was downregulated in BGC-823 cells using three specific siRNAs targeting *CASC19* (si*CASC19*). Based on the interference efficiency of these specific siRNAs, si-*CASC19*-2 was used in the following experiments (Figure 6B). The Cell Counting Kit-8 (CCK-8) assay showed that *CASC19* knockdown significantly inhibited cell proliferation in BGC-823 cells (Figure 6C). Furthermore, the colony formation assay showed that the number and size of all colonies in the *CASC19* knockdown group were significantly reduced compared with the control group (Figure 6D), indicating that *CASC19* also promotes anchorage-independent growth of GC cells. Additionally, *CASC19* knockdown markedly suppressed migration and invasion of BGC-823 cells (Figure 6E and 6F). These findings demonstrate that knockdown of *CASC19* inhibits proliferation and metastasis in GC cells.

## DISCUSSION

Accumulating evidence has revealed that aberrantly expressed lncRNAs contribute to the development and progression of AGC [12–14]. Multiple studies also confirmed that several lncRNAs such as *HOTAIR*, *H19* and *MALAT1* have a pivotal function in the carcinogenesis of AGC and are expected to become therapeutic targets for its treatment [21–27]. Here, we successfully performed an innovative genomic analysis method that combined differential lncRNA expression profiling with WGCNA to identify key lncRNAs associated with development and progression of AGC. Remarkably, we found that *CASC19* was upregulated in AGC tissues and this phenomenon was highly correlated with higher clinicopathologic parameters and worse prognosis in AGC patients.

*CASC19*, also known as *CARLo-6* and *LINC01245*, is a long intergenic non-coding RNA of 324 bp in length encoded on chromosome 8q24.21 [28]. This region lacks protein-coding genes, so it has been called a 'gene desert' or 'ncRNA oasis' [29]. Previous genome-wide association studies (GWAS) have demonstrated multiple genetic variants in the 8q24.21 region that significantly increase the susceptibility of some cancers, such as colorectal, prostate, and breast cancer [28, 30–33]. Sotelo et al. [30] reported that cancer-associated single nucleotide polymorphism (SNP) rs6983267, which regulates enhancer activity in this region, can regulate the transcription of the nearest annotated gene, the proto-oncogene *MY*C. Kim et al. [28] found that the SNP rs6983267 regulates the expression of *CCAT1* in this region through long-range interaction with its promoter in colorectal cancer (CRC), while Wasserman et al. [29] showed that rs6983267 correlates with *MYC* expression in prostate cancer. Based on these findings, we hypothesis that *CASC19*, along with other lncRNAs in this chromosomal region, may also be critically involved in the progression of cancer.

*CASC19* has been reported to enhance proliferation of CRC cells, and to promote cell migration by acting as a competitive endogenous RNA that induces hyaluronidase 1 expression by sponging miR-140-5p [34]. GWAS studies have showed that upregulation of the *CASC19* gene containing the SNP rs138042437 greatly increases prostate cancer susceptibility [35–37]. Meanwhile, Ozawa et al. [31] described that *CASC19* was significantly upregulated in CRC specimens compared with normal colonic tissue. However, few studies have investigated the role of *CASC19* in AGC. In this work, we demonstrated that *CASC19* expression is upregulated in AGC and is positively associated with pathologic T stage, TNM stage, and LN metastasis. These findings suggest that *CASC19* overexpression may be related to carcinogenesis and progression of AGC. Additionally, *CASC19* overexpression correlated with poor overall survival in AGC patients and was an independent prognostic factor for overall survival in AGC. Altogether, our data suggest that *CASC19* can be considered an oncogene and might be a novel candidate biomarker for the prognosis and therapy of AGC.

This hypothesis was substantiated by in vitro experiments that showed that *CASC19* was upregulated in human GC cell lines, and that si-RNA-mediated knockdown inhibited proliferation, anchorage-independent growth, migration, and invasion in the BGC-823 cell line. Interestingly, *CASC19* seems to affect more on migration and invasion than proliferation in BGC-823 cell line. This may be due to the fact that the downstream genes affected by *CASC19* knockdown are preferentially associated to cell migration and invasion. However, further RNA-seq and pathway analyses are required to validate the changes in their expression caused by *CASC19* knockdown. Therefore, we will verify this in the next mechanism study.

We performed GSEA analysis to elucidate potential functions of *CASC19*. Interestingly, for the *CASC19* high-expression phenotype most gene sets were significantly enriched in the ‘cancer-related’ and ‘classical signaling’ pathways. Within these categories, *CASC19* high-expression samples were significantly enriched in ‘focal adhesion’, ‘neuroactive ligand receptor interaction’, and ‘regulation of actin cytoskeleton’ pathways. Focal adhesion are macromolecular assemblies associated to the plasma membrane that mediate strong adhesion to the extracellular matrix and influence signaling pathways closely related to cell differentiation, proliferation, and invasion [38–40]. Xu et al. [41] found that GSEA’s ‘neuroactive ligand receptor interaction’ pathway was highly upregulated and contributed to the oncogenesis of endometrial carcinoma, and the influence of this pathway has been recently reported also for AGC [42–44]. Many studies have also proved that changes in the actin cytoskeleton contribute to cancer cell migration and invasion [45–47]. Meanwhile, GSEA identified that *CASC19* may participate in the carcinogenesis of AGC by regulating the MAPK, calcium, wnt and insulin signaling pathways. Indeed, previous studies demonstrated that the MAPK and wnt signaling pathway are critical for GC cell proliferation, apoptosis, and metastasis [48–51]. Huang et al. [52] indicated that a positive feedback loop between the calcium signaling pathway and mitochondrial fission promotes autophagy in hepatocellular carcinoma cells. Regarding GC, several calcium signaling alterations have been reported to contribute to its progression [53–55]. Meanwhile, the insulin signaling pathway is primarily involved in the pathogenesis of human obesity and type 2 diabetes [56], conditions that are known to facilitate the development of several cancers [57, 58], including GC [59].

The major limitation of this study is that the specific mechanisms by which *CASC19* overexpression may contribute to AGC have not been fully explored. In future studies we will use clinical data and in vivo experiments to verify and further explore upstream and downstream interactions of *CASC19* in GC.

## CONCLUSIONS

Our study suggested that *CASC19* is involved in the progression of AGC, probably by regulating the MAPK, calcium, wnt, and insulin signaling pathways, and therefore arises as a novel prognostic indicator and potential therapeutic target. Further experiments are needed to elucidate the molecular mechanisms affected by *CASC19* in the carcinogenesis of AGC, which may lead to more effective treatment strategies.

## MATERIALS AND METHODS

### Study design and data collection

GC patient RNA sequencing data and clinical information were downloaded from TCGA (https://portal.gdc.cancer.gov/) database on April 30, 2019 using the Data Transfer Tool. We obtained 407 samples in total (375 tumor and 32 normal tissue samples; data type: FPKM; platform: IlluminaHiseq; project ID: TCGA-STAD) to identify differentially expressed lncRNAs. Afterwards, GC tissue samples with complete clinical data were selected to acquire hub lncRNAs. Next, patients with pT2-4aN0-3M0-1 and complete survival information were further collected for subsequent analysis, and 351 eligible patients were finally retained (Table 1).

### Data pre-processing and DElncRNAs screening

In this step, we applied the GENCODE gene annotation file (Release 30/GRCh38.p12) (https://www.gencodegenes.org/human/) to re-annotate probes with corresponding lncRNAs [60]. For multiple probes corresponding to an identical lncRNA, the average was calculated as its final expression value. The “edgeR” R package (version 3.24.0) was employed to identify DElncRNAs between GC tissues and non-tumor tissues [61]. A |log2 FC| > 2.0 and a FDR < 0.01 were set as thresholds.

### Weighted gene co-expression network construction

To evaluate potential contributions of lncRNAs to the molecular mechanism of AGC, WGCNA was carried out to construct an lncRNA co-expression network using the “WGCNA” R package (version 1.66) [62]. First, the goodSamplesGenes function was used to remove lncRNAs and samples with missing values, and the sample hierarchical clustering-pruning method was applied to remove outlier samples before building a network [63]. Second, the adjacency matrix was constructed by calculating the connection strength of each pair of genes according to the following formula: a_ij_ = power (S_ij_, β) = |S_i, j_|^β^, where a_ij_ represents the adjacency function between gene i and gene j, and β is the soft thresholding power [64]. Third, the adjacency matrix was converted to a TOM by calculating the degree of association between lncRNAs [65]. The TOM_i, j_ between lncRNA i and lncRNA j was calculated by the following formula: TOM_i, j_ = ∑_u_a_iu_a_uj_ + a_ij_ / [min (k_i_, k_j_) + 1− a_ij_], where ∑_u_a_iu_a_uj_ denotes the sum of the products of the adjacent coefficients of the nodes in which the lncRNA i and the lncRNA j are connected in common, and k_i_ = ∑_u_a_iu_ signifies the sum of the adjacency coefficients of all nodes to which lncRNA i is individually connected. Next, TOM was converted into a dissimilarity TOM (dissTOM_i, j_ = 1− TOM_i, j_), and the dynamic Tree Cut method was used to establish a hierarchical clustering tree according to the similarity and dissimilarity matrices (TOM_i, j_ and dissTOM_i, j_) [66]. The minimum number of lncRNAs in each clustering tree was set as 60, and the threshold for similar modules to be merged was set to 0.5 [67].

### Identification of clinical significant modules

In order to find lncRNAs significantly related to AGC, two approaches were performed in this study. First, module eigengenes (MEs) were calculated according to the overall level of expression of all lncRNAs in the module. In our study, the relationship between MEs and pathologic TNM stage was applied to screen the most significant module. Second, GS was defined as the value of the correlation coefficient between each lncRNA gene’s expression profile and every clinical trait, and MS was defined as the average GS for all lncRNAs contained in the module. In general, a higher MS indicated a higher correlation between this module and the phenotype. Third, MM of each lncRNA in the module was defined as the correlation coefficient between each lncRNA and the ME. The module with the highest correlation to pathologic TNM stage was chosen for further analysis. LncRNA with the highest MM and GS in the selected module was considered as a hub lncRNA and further analyzed.

### Hub lncRNAs screening

We selected the module with the highest correlation with pathologic TNM staging for further analysis. “VennDiagram” R package (version 1.6.20) was used to to screen out relevant lncRNAs among those in the selected module and the previously obtained DElncRNAs [68]. We considered overlapping lncRNA with the highest MM and GS as a hub lncRNAs, and those was selected for deeper analysis and validation.

### Correlation with hub lncRNAs expression and clinicopathologic parameters

To investigate the relationship between hub lncRNA expression and clinicopathologic parameters, we comprehensively analyzed the differential expression of hub lncRNAs in relation to various clinicopathologic parameters including age, gender, histology, pathological TNM stage, pathological T stage, LN metastasis, and distant metastasis. Wilcoxon-Mann-Whitney test and logistic regression were conducted using R software (version 3.5.1). ORs and the corresponding 95% CIs were assessed and a two tailed *P* < 0.05 was considered statistically significant.

### Survival outcomes and cox regression analysis

X-tile software (version 3.6.1) was used to calculate the optimal cutoff value for hub lncRNA expression according to the maximum χ^2^ test and the minimum *P*-value [69]. Next, AGC patients were divided into high- and low- expression groups based on the optimal cutoff value for hub lncRNA. Survival analysis was performed by the Kaplan–Meier method with log-rank test. Univariate and multivariate analyses used the Cox proportional hazards regression model to estimate risk factors for overall survival, and results were presented as HRs with 95% CIs. Statistical analyses were performed through SPSS 23.0 (SPSS INC., Chicago, IL, USA). All statistical tests were two-sided, and *P* < 0.05 was considered statistically significant.

### GSEA

GSEA (http://software.broadinstitute.org/gsea/index.jsp) was performed to elucidate potential functions of hub lncRNA [70, 71]. AGC samples were divided into a high-expression group and a low-expression group depending on the expression level of hub lncRNA. We chose the annotated gene set of c2.cp.kegg.v6.2.symbols.gmt as a reference and selected the expression level of hub lncRNA as a phenotype label. The number of permutations of GSEA was set as 1000 times for each analysis. NOM *P* < 0.05, FDR less than 25%, and gene size more than 50 were chosen as the thresholds.

### Cell culture and transfection

Human GC cell lines (AGS, BGC-823, MGC-803, and HGC-27) and a normal gastric mucosal epithelial cell line (GES-1) were purchased from the Chinese Academy of Sciences (Shanghai, China). Cells were cultured in Dulbecco’s modified Eagle’s medium (DMEM) supplemented with 10% fetal bovine serum (FBS) (Gibco) at 37 °C with 5% CO_2_. Three *CASC19* small interfering RNAs (siRNAs, si-*CASC19*) and a scrambled negative control siRNA (si-NC) were designed and synthesized by GenePharma (Shanghai, China). The siRNA sequences are shown in Supplementary Table 4. Transfections were conducted using Lipofectamine 2000 (Invitrogen) according to the manufacturer's instructions. Cells were harvested 48 hours after transfection for subsequent experiments.

### qRT-PCR

Total RNA was extracted using Trizol (SuperfecTRI, Shanghai, China) in accordance with the manufacturer’s instructions. qRT-PCR was performed with SYBR Green Master Mixture (Takara, Dalian, China). Primer sequences are available in Supplementary Table 4. *GAPDH* was used as the endogenous control. Relative *CASC19* expression levels were calculated using the 2^−ΔΔct^ method.

### Cell proliferation assay

The CCK-8 kit (Dojindo, Japan) was used to detect the effect of *CASC19* knockdown on GC cell proliferation. BGC-823 cells were seeded into 96-well plates at a concentration of 2,000 cells/well. At 1, 2, 3, 4, and 5 days post-seeding CCK-8 was added to wells for 2 hours and its absorbance measured at 450 nm to estimate cell numbers.

### Colony formation assay

BGC-823 cells were seeded into 6-well plates at a concentration of 1,000 cells/well 3 days posttransfection. Cells were cultured for 10 days, and then fixed with 4% paraformaldehyde for 30 minutes, washed once with PBS, and stained with 0.1% crystal violet for 10 minutes. Colony formation was quantified using Image J software (National Institutes of Health, Bethesda, MD, USA).

### Cell migration and invasion assays

Cell migration and invasion assays were carried out using Transwell chamber inserts (8-μm pores; Corning, NY, USA) in a 24-well plate. BGC-823 cells (1 × 10^5^) were suspended in serum-free medium and seeded in Matrigel-coated chambers and in non-coated chambers for invasion and migration assays, respectively. DMEM containing 30% FBS was added to the lower chamber and plates were maintained in a 37 °C incubator for 24 hours. Next, migrating/invading cells were fixed with methanol and stained with 0.1% crystal violet, and counted in three randomly selected fields using Image J software.

### Statistical analysis

All data were analyzed with GraphPad Prism 7 software (San Diego, CA, USA). Differences between two groups were assessed by Student’s t-test. All experiments were repeated three times and average results were calculated. A two-sided *P* < 0.05 was considered statistically significant.

### Ethical approval

This study was approved by the Institute’s Research Ethics Committee of The 940^th^ Hospital of Joint Logistics Support Force of Chinese People’s Liberation Army (2019KYLL010).

## Supplementary Material

Supplementary Tables

Supplementary Table 1
