# Supplementary material for: Long non-coding RNA CASC19 is associated with the progression and prognosis of advanced gastric cancer
Source: Aging (Albany NY). 2019 Aug 15;11(15):5829–47. doi: 10.18632/aging.102190 (PMC6710062; doi:10.18632/aging.102190)
Supplement: Supplementary Tables [file aging-11-102190-s002.pdf]

SUPPLEMENTARY TABLES

Please browse Full Text version to see the data of Supplementary Table 1:

Supplementary Table 1. All DElncRNAs between GC tissues and non-tumor tissues in this study.

Supplementary Table 2. Eleven overlapping lncRNAs between DElncRNAs and the lncRNAs in the brown module.

Gene symbol	logFC	logCPM	PValue	FDR
AC108463.3	2.617232	4.654981	4.05E-26	1.01E-23
AL135924.2	-2.44865	4.100404	4.09E-16	2.47E-14
AC008114.1	2.425031	3.619495	1.22E-12	3.92E-11
AL512413.1	2.249373	4.990477	4.39E-11	1.02E-09
MYB-AS1	3.015976	3.010859	4.23E-10	8.08E-09
AC012467.1	2.293675	3.875899	1.03E-09	1.77E-08
AP005233.2	2.295633	6.175382	5.26E-09	7.62E-08
KRT7-AS	2.427818	7.166851	1.40E-08	1.84E-07
CASC19	2.273281	6.647498	2.95E-08	3.58E-07
AC010998.1	3.545427	2.931989	3.96E-07	3.51E-06
AC012668.3	2.233674	3.285144	3.76E-05	1.92E-04

Supplementary Table 3. Eight representative pathways enriched in AGC samples with high CASC19 expression from GSEA.

Name	Size	ES	NES	NOM p-val	FDR q-val
Pathways in cancer	321	0.435032	1.636691	0.024845	0.107062
Meuroactive ligand receptor interaction	270	0.438235	1.748006	0.008403	0.091728
MAPK signaling pathway	265	0.482662	1.903347	0	0.092143
Regulation of actin cytoskeleton	211	0.410933	1.603861	0.045643	0.107686
Focal adhesion	197	0.504511	1.724	0.041152	0.09182
Calcium signaling pathway	176	0.532904	2.04586	0	0.059877
Wnt signaling pathway	149	0.477498	1.757245	0.004	0.099863
Insulin signaling pathway	137	0.484351	1.783265	0.00409	0.104717

Supplementary Table 4. Sequences used for silencing and qRT-PCR in the present study.

Genes	Sequence 5′-3′
si-CASC19-1	5′-CAGCATTTGCCATACTACATT-3′
si-CASC19-2	5′-CAGCACAATGATGGAAGGCTT-3′
si-CASC19-3	5′-CTGCATGCTTCTGATGTGAGT-3′
si-NC	5′-TTCTCCGAACGTGTCACGT-3′
CASC19	F: 5′-TTGGAGTGCCTGGGTTAGA-3′<br>R: 5′-CTGTCCTTGCCAGTGTCTT-3′
GAPDH	F: 5′-TGACTTCAACAGCGACACCCA-3′<br>R: 5′-CACCCTGTTGCTGTAGCCAAA-3′
