# Supplementary material for: Long non-coding RNA CASC19 is associated with the progression and prognosis of advanced gastric cancer
Source: Aging (Albany NY). 2019 Aug 15;11(15):5829–47. doi: 10.18632/aging.102190 (PMC6710062; doi:10.18632/aging.102190)
Supplement: Supplementary Table 1 [file aging-11-102190-s001.docx]

**Supplementary Table 1. All DElncRNAs between GC tissues and non-tumor tissues in this study.**

| **Gene Symbol** | **logFC** | **logCPM** | **PValue** | **FDR** |
| --- | --- | --- | --- | --- |
| AC115619.1 | -5.34404 | 5.283214 | 2.61E-26 | 6.82E-24 |
| AP003037.1 | -5.24236 | 2.541494 | 7.48E-20 | 7.73E-18 |
| AC133485.2 | -4.8682 | 3.586944 | 9.47E-21 | 1.15E-18 |
| HCG22 | -4.63224 | 7.33354 | 9.07E-37 | 1.05E-33 |
| LINC02471 | -4.50192 | 3.109005 | 5.04E-29 | 1.81E-26 |
| LHX5-AS1 | -4.48872 | 2.231171 | 1.75E-24 | 3.26E-22 |
| AL391361.3 | -4.44976 | 2.601478 | 2.96E-23 | 4.83E-21 |
| AC022081.1 | -4.25705 | 2.142852 | 3.32E-28 | 1.15E-25 |
| AC084030.1 | -4.09978 | 2.233503 | 2.95E-20 | 3.28E-18 |
| LINC00955 | -4.02188 | 5.193065 | 5.13E-22 | 7.04E-20 |
| AC007920.1 | -3.88807 | 3.333789 | 2.51E-25 | 5.46E-23 |
| AC018742.1 | -3.88483 | 3.422831 | 1.09E-38 | 1.62E-35 |
| AC244230.1 | -3.80482 | 2.160979 | 1.18E-15 | 6.60E-14 |
| AC103563.7 | -3.78781 | 4.097518 | 1.28E-29 | 5.13E-27 |
| AC024597.1 | -3.68291 | 2.823577 | 3.43E-21 | 4.53E-19 |
| AC011374.1 | -3.66566 | 4.175683 | 6.19E-28 | 2.08E-25 |
| AC091230.1 | -3.63783 | 3.062728 | 4.73E-35 | 3.80E-32 |
| AC008268.1 | -3.63487 | 4.134457 | 3.96E-17 | 2.87E-15 |
| PGM5-AS1 | -3.60715 | 6.763274 | 2.24E-27 | 6.88E-25 |
| IL12A-AS1 | -3.58011 | 5.098212 | 7.43E-61 | 7.75E-57 |
| LINC02200 | -3.5674 | 2.305408 | 7.80E-20 | 7.91E-18 |
| C5orf66-AS1 | -3.52552 | 7.326368 | 8.28E-36 | 8.64E-33 |
| AC009123.1 | -3.48855 | 3.179994 | 1.59E-15 | 8.47E-14 |
| LINC02560 | -3.45173 | 5.21287 | 1.65E-23 | 2.73E-21 |
| AL136982.6 | -3.42277 | 3.210656 | 8.16E-33 | 5.32E-30 |
| AC110491.1 | -3.33217 | 3.969544 | 5.14E-19 | 4.84E-17 |
| AC103563.2 | -3.31139 | 4.530001 | 1.61E-10 | 3.37E-09 |
| MIR205HG | -3.30686 | 8.495812 | 2.56E-08 | 3.18E-07 |
| AP001107.5 | -3.29785 | 6.179748 | 3.20E-46 | 8.36E-43 |
| AL121796.1 | -3.26464 | 2.252291 | 6.56E-14 | 2.64E-12 |
| AL589986.2 | -3.24958 | 2.806475 | 5.96E-18 | 4.83E-16 |
| AL359979.1 | -3.23266 | 2.485075 | 6.20E-17 | 4.26E-15 |
| LINC02490 | -3.22814 | 3.296656 | 6.08E-16 | 3.49E-14 |
| LINC00330 | -3.21444 | 5.09117 | 3.90E-21 | 5.09E-19 |
| PCAT18 | -3.18345 | 5.382357 | 1.27E-20 | 1.49E-18 |
| AC083902.1 | -3.15316 | 2.682584 | 7.56E-18 | 5.98E-16 |
| AC051619.8 | -3.13933 | 2.812838 | 1.25E-20 | 1.48E-18 |
| AC012055.1 | -3.13841 | 2.780918 | 9.58E-15 | 4.45E-13 |
| CERS3-AS1 | -3.10476 | 3.2499 | 6.93E-26 | 1.65E-23 |
| LINC00844 | -3.09832 | 2.136785 | 1.97E-25 | 4.37E-23 |
| CISTR | -3.07147 | 2.394639 | 6.03E-21 | 7.59E-19 |
| AC021242.2 | -3.07075 | 3.126575 | 5.39E-15 | 2.65E-13 |
| AC006007.1 | -3.03424 | 2.965978 | 2.47E-10 | 4.94E-09 |
| AL023754.1 | -2.98302 | 3.221019 | 8.39E-11 | 1.84E-09 |
| AC055874.1 | -2.96469 | 2.665744 | 3.31E-13 | 1.19E-11 |
| LRRC3-DT | -2.96061 | 3.293718 | 1.38E-29 | 5.32E-27 |
| LINC01497 | -2.95966 | 2.66273 | 3.09E-15 | 1.60E-13 |
| AP003500.1 | -2.95734 | 2.80473 | 3.95E-12 | 1.11E-10 |
| AL033380.1 | -2.95476 | 2.225 | 5.59E-13 | 1.93E-11 |
| AC097103.1 | -2.95355 | 2.51908 | 8.39E-28 | 2.65E-25 |
| LINC02487 | -2.93933 | 7.603164 | 9.99E-23 | 1.49E-20 |
| LINC01697 | -2.93225 | 5.652385 | 1.27E-30 | 5.54E-28 |
| ADAMTS9-AS1 | -2.92929 | 6.549341 | 1.43E-49 | 7.49E-46 |
| UBXN10-AS1 | -2.91085 | 6.097381 | 5.43E-32 | 2.84E-29 |
| LINC00671 | -2.85366 | 4.288629 | 3.68E-35 | 3.20E-32 |
| AC105398.1 | -2.83815 | 1.998948 | 7.49E-16 | 4.27E-14 |
| AL021026.1 | -2.83515 | 3.446401 | 6.65E-21 | 8.27E-19 |
| HAND2-AS1 | -2.81726 | 10.116 | 4.16E-16 | 2.50E-14 |
| AL079303.1 | -2.80212 | 4.669642 | 2.93E-09 | 4.51E-08 |
| LINC02404 | -2.80179 | 8.141103 | 4.05E-10 | 7.79E-09 |
| LINC01214 | -2.79312 | 2.91503 | 2.62E-11 | 6.41E-10 |
| MIR1-1HG-AS1 | -2.78272 | 6.895835 | 3.45E-19 | 3.40E-17 |
| AL162511.1 | -2.75747 | 3.414613 | 5.39E-12 | 1.49E-10 |
| LINC02557 | -2.74197 | 2.078702 | 2.53E-12 | 7.41E-11 |
| LINC02268 | -2.71457 | 4.599378 | 5.83E-15 | 2.84E-13 |
| LINC02310 | -2.69817 | 2.364364 | 5.32E-10 | 9.90E-09 |
| CALML3-AS1 | -2.69726 | 6.524251 | 5.73E-15 | 2.81E-13 |
| AC244205.1 | -2.6938 | 5.165895 | 2.22E-22 | 3.13E-20 |
| LINC01100 | -2.68771 | 2.245046 | 7.26E-10 | 1.31E-08 |
| AF165147.1 | -2.68356 | 5.482514 | 8.23E-40 | 1.43E-36 |
| AC108861.1 | -2.68064 | 2.238161 | 4.40E-14 | 1.82E-12 |
| AC079467.1 | -2.67981 | 2.670296 | 4.43E-15 | 2.24E-13 |
| LINC01336 | -2.67601 | 5.476816 | 1.44E-31 | 7.14E-29 |
| CDKN2B-AS1 | -2.67102 | 7.199477 | 6.64E-28 | 2.17E-25 |
| AC092422.1 | -2.66541 | 3.534071 | 6.25E-23 | 9.60E-21 |
| LINC00332 | -2.65928 | 2.708039 | 3.09E-10 | 6.10E-09 |
| AP001360.2 | -2.65452 | 2.007754 | 1.18E-09 | 2.00E-08 |
| AP000721.2 | -2.64835 | 3.585795 | 1.86E-25 | 4.22E-23 |
| DSG1-AS1 | -2.62636 | 5.659396 | 3.07E-11 | 7.42E-10 |
| AC005906.2 | -2.62164 | 2.877846 | 3.97E-11 | 9.32E-10 |
| AP001554.1 | -2.61332 | 3.306022 | 4.08E-10 | 7.85E-09 |
| LL22NC03-63E9.3 | -2.61238 | 3.183981 | 4.53E-16 | 2.66E-14 |
| LINC02106 | -2.59448 | 4.45216 | 4.32E-19 | 4.14E-17 |
| AC092691.1 | -2.59069 | 2.939676 | 2.48E-13 | 9.18E-12 |
| AC002398.2 | -2.58459 | 4.986165 | 1.16E-13 | 4.49E-12 |
| HNF4A-AS1 | -2.5767 | 6.322483 | 1.04E-32 | 6.41E-30 |
| AC023090.1 | -2.55959 | 7.06755 | 5.34E-17 | 3.69E-15 |
| AC087379.2 | -2.55937 | 2.930088 | 3.20E-08 | 3.84E-07 |
| NALT1 | -2.54975 | 6.440441 | 1.01E-29 | 4.22E-27 |
| AL513217.1 | -2.54722 | 3.978307 | 3.12E-14 | 1.33E-12 |
| PTCHD1-AS | -2.5385 | 2.422571 | 1.86E-12 | 5.60E-11 |
| AC008406.3 | -2.53812 | 4.359565 | 3.26E-27 | 9.71E-25 |
| MPPED2-AS1 | -2.53785 | 3.004763 | 1.71E-11 | 4.33E-10 |
| AC005722.3 | -2.53516 | 3.334981 | 8.39E-15 | 4.00E-13 |
| AC087521.1 | -2.53294 | 4.9332 | 1.07E-37 | 1.39E-34 |
| AC112236.1 | -2.53118 | 3.520446 | 2.12E-12 | 6.27E-11 |
| LINC01055 | -2.53076 | 4.585645 | 1.51E-24 | 2.86E-22 |
| AP001065.1 | -2.53019 | 4.884666 | 3.04E-32 | 1.67E-29 |
| AC104407.1 | -2.52928 | 2.427354 | 3.05E-08 | 3.67E-07 |
| AP000802.1 | -2.50636 | 3.503251 | 1.06E-18 | 9.26E-17 |
| LINC00551 | -2.50367 | 2.6634 | 1.05E-13 | 4.11E-12 |
| SOX21-AS1 | -2.49389 | 9.125054 | 2.03E-11 | 5.05E-10 |
| PART1 | -2.48587 | 8.855145 | 3.88E-18 | 3.24E-16 |
| AC007182.1 | -2.47993 | 4.991355 | 9.90E-14 | 3.90E-12 |
| AC121764.1 | -2.46835 | 2.84565 | 8.06E-05 | 0.000371 |
| LINC00982 | -2.46585 | 9.290193 | 3.52E-24 | 6.13E-22 |
| LINC01634 | -2.45743 | 3.156402 | 4.23E-23 | 6.79E-21 |
| AL135924.2 | -2.44865 | 4.100404 | 4.09E-16 | 2.47E-14 |
| AC025280.1 | -2.44635 | 3.755701 | 7.82E-26 | 1.81E-23 |
| AC114400.1 | -2.44345 | 2.0349 | 2.78E-09 | 4.31E-08 |
| LINC00682 | -2.43486 | 2.612215 | 9.01E-15 | 4.24E-13 |
| MRGPRF-AS1 | -2.42774 | 4.076772 | 3.77E-20 | 4.01E-18 |
| AC007221.1 | -2.41972 | 2.352642 | 1.55E-10 | 3.26E-09 |
| AC092834.1 | -2.39733 | 3.916784 | 1.82E-12 | 5.49E-11 |
| AC093159.1 | -2.39028 | 3.230434 | 1.53E-20 | 1.78E-18 |
| AC036108.3 | -2.38994 | 7.572821 | 4.46E-18 | 3.70E-16 |
| LINC02031 | -2.38747 | 2.757555 | 2.57E-08 | 3.18E-07 |
| AL359633.2 | -2.38693 | 2.30225 | 2.08E-12 | 6.23E-11 |
| AC053503.4 | -2.38598 | 5.028793 | 1.84E-09 | 3.01E-08 |
| LINC00365 | -2.38395 | 7.704629 | 1.63E-17 | 1.26E-15 |
| AC110619.1 | -2.37586 | 5.597255 | 4.18E-14 | 1.75E-12 |
| AC103740.1 | -2.36847 | 7.276955 | 6.81E-23 | 1.03E-20 |
| AC233266.2 | -2.36494 | 4.10372 | 1.21E-16 | 8.02E-15 |
| LINC00582 | -2.36002 | 4.163956 | 3.65E-20 | 3.95E-18 |
| AP003396.3 | -2.35939 | 2.68427 | 4.50E-18 | 3.70E-16 |
| ARHGEF26-AS1 | -2.35895 | 5.60793 | 3.66E-19 | 3.57E-17 |
| RBMS3-AS3 | -2.35587 | 3.686679 | 1.03E-22 | 1.51E-20 |
| GS1-594A7.3 | -2.35466 | 2.523364 | 3.44E-09 | 5.26E-08 |
| LINC00284 | -2.34701 | 3.923295 | 4.74E-09 | 6.96E-08 |
| AC002546.1 | -2.34699 | 3.560151 | 1.37E-15 | 7.51E-14 |
| AC099684.2 | -2.34564 | 5.476449 | 9.20E-11 | 2.01E-09 |
| AC116407.1 | -2.34304 | 6.033134 | 3.85E-42 | 8.04E-39 |
| AC126768.2 | -2.33888 | 3.703631 | 1.37E-14 | 6.18E-13 |
| AL139393.1 | -2.33809 | 2.611215 | 4.80E-12 | 1.34E-10 |
| GAS1RR | -2.33498 | 5.677402 | 4.12E-21 | 5.31E-19 |
| AC105389.3 | -2.33189 | 1.973301 | 2.88E-06 | 2.06E-05 |
| PDZRN3-AS1 | -2.32652 | 2.562445 | 2.51E-14 | 1.07E-12 |
| DIO2-AS1 | -2.32052 | 2.493636 | 1.30E-10 | 2.76E-09 |
| AL161457.1 | -2.31663 | 3.119546 | 7.36E-12 | 1.98E-10 |
| AC093607.1 | -2.30491 | 3.401274 | 9.63E-12 | 2.53E-10 |
| ADAMTS9-AS2 | -2.29261 | 7.122341 | 5.13E-26 | 1.25E-23 |
| LINC02177 | -2.28983 | 4.739764 | 1.52E-14 | 6.84E-13 |
| FRMD6-AS2 | -2.28935 | 3.2913 | 2.98E-13 | 1.08E-11 |
| IL20RB-AS1 | -2.2882 | 2.769066 | 1.93E-14 | 8.44E-13 |
| MIR1-1HG | -2.28485 | 2.915881 | 5.84E-10 | 1.08E-08 |
| AC084880.3 | -2.28455 | 2.729997 | 6.98E-13 | 2.37E-11 |
| CHL1-AS2 | -2.284 | 3.390938 | 2.14E-13 | 7.94E-12 |
| AC092667.1 | -2.27995 | 3.369347 | 6.93E-13 | 2.36E-11 |
| AF001548.1 | -2.27885 | 6.306116 | 7.47E-13 | 2.51E-11 |
| AC005532.1 | -2.27337 | 6.100504 | 1.35E-15 | 7.41E-14 |
| AC104024.1 | -2.27163 | 3.84394 | 3.67E-20 | 3.95E-18 |
| LINC02158 | -2.27045 | 4.089295 | 9.49E-31 | 4.31E-28 |
| FLG-AS1 | -2.2675 | 5.860217 | 2.32E-20 | 2.66E-18 |
| NCOA7-AS1 | -2.25413 | 3.308526 | 4.24E-17 | 3.03E-15 |
| MEF2C-AS1 | -2.24297 | 5.66145 | 1.45E-26 | 4.08E-24 |
| AP003548.1 | -2.23488 | 3.021095 | 3.37E-17 | 2.48E-15 |
| AL357153.1 | -2.23095 | 4.265535 | 1.32E-12 | 4.19E-11 |
| BVES-AS1 | -2.21467 | 4.896798 | 1.54E-15 | 8.31E-14 |
| AL731684.1 | -2.21389 | 2.140874 | 7.24E-09 | 1.01E-07 |
| PWAR6 | -2.2134 | 7.490288 | 1.12E-21 | 1.53E-19 |
| FALEC | -2.19718 | 4.287736 | 3.42E-24 | 6.06E-22 |
| AC005019.2 | -2.19407 | 2.342994 | 6.67E-05 | 0.000315 |
| MKX-AS1 | -2.19369 | 2.926549 | 3.16E-10 | 6.21E-09 |
| AC012558.1 | -2.18967 | 2.061726 | 3.51E-09 | 5.35E-08 |
| AC027807.2 | -2.17796 | 3.044941 | 1.06E-14 | 4.88E-13 |
| TBX5-AS1 | -2.1764 | 4.623014 | 1.90E-07 | 1.84E-06 |
| AC012085.2 | -2.17387 | 5.167848 | 2.10E-24 | 3.84E-22 |
| AC124312.4 | -2.16439 | 3.205136 | 4.54E-14 | 1.87E-12 |
| AC116337.3 | -2.16131 | 3.318723 | 1.24E-14 | 5.66E-13 |
| AC116312.1 | -2.15086 | 2.073554 | 4.45E-09 | 6.58E-08 |
| TMEM220-AS1 | -2.15073 | 6.841853 | 1.61E-31 | 7.63E-29 |
| AL353997.2 | -2.14091 | 3.917916 | 6.89E-14 | 2.76E-12 |
| AC079305.3 | -2.13028 | 2.698602 | 4.99E-15 | 2.48E-13 |
| LINC01395 | -2.12424 | 3.386825 | 3.66E-08 | 4.36E-07 |
| AL033384.1 | -2.12302 | 5.547127 | 1.84E-13 | 6.85E-12 |
| LINC01082 | -2.12268 | 6.166614 | 3.05E-17 | 2.26E-15 |
| AC107067.1 | -2.12132 | 3.491596 | 3.79E-17 | 2.77E-15 |
| AC093702.1 | -2.11924 | 3.512656 | 8.94E-08 | 9.41E-07 |
| AC007126.1 | -2.11591 | 2.625737 | 2.24E-08 | 2.83E-07 |
| AL512274.1 | -2.10588 | 9.308842 | 5.22E-19 | 4.87E-17 |
| AC015845.2 | -2.09587 | 3.085213 | 1.70E-14 | 7.52E-13 |
| BARX1-DT | -2.0905 | 6.164125 | 1.07E-12 | 3.47E-11 |
| AC074033.1 | -2.08809 | 4.793591 | 7.61E-20 | 7.78E-18 |
| AC246787.2 | -2.08589 | 3.89454 | 4.68E-10 | 8.86E-09 |
| OSTN-AS1 | -2.0854 | 3.819448 | 8.80E-13 | 2.88E-11 |
| LINC00940 | -2.08396 | 3.683773 | 1.26E-10 | 2.68E-09 |
| AP000915.1 | -2.08134 | 2.387676 | 8.78E-13 | 2.88E-11 |
| LINC01527 | -2.07415 | 3.328101 | 7.06E-05 | 0.000331 |
| AC011472.4 | -2.07095 | 5.10077 | 7.55E-34 | 5.63E-31 |
| NKAIN3-IT1 | -2.07013 | 6.103752 | 3.78E-11 | 8.90E-10 |
| VSTM2A-OT1 | -2.06953 | 2.208397 | 1.30E-07 | 1.31E-06 |
| AC090531.1 | -2.06733 | 2.222719 | 1.10E-07 | 1.13E-06 |
| AC226101.1 | -2.06232 | 2.141147 | 7.12E-10 | 1.29E-08 |
| AC093583.1 | -2.04856 | 5.867927 | 6.81E-13 | 2.33E-11 |
| AC015908.3 | -2.04805 | 5.42399 | 5.33E-23 | 8.30E-21 |
| AC009229.1 | -2.04752 | 2.294219 | 4.30E-08 | 5.02E-07 |
| AC026774.1 | -2.04557 | 2.13096 | 3.91E-06 | 2.69E-05 |
| AP002761.4 | -2.04521 | 8.687889 | 1.27E-16 | 8.27E-15 |
| NCAM1-AS1 | -2.04511 | 2.777318 | 3.72E-10 | 7.18E-09 |
| LINC00163 | -2.04445 | 3.547118 | 1.92E-09 | 3.11E-08 |
| AC138430.1 | -2.03663 | 3.321381 | 1.66E-13 | 6.20E-12 |
| AC008808.2 | -2.03077 | 5.676407 | 2.35E-15 | 1.23E-13 |
| AC104699.1 | -2.02827 | 5.640905 | 9.06E-19 | 8.15E-17 |
| AL357054.4 | -2.02588 | 7.182474 | 3.66E-26 | 9.31E-24 |
| CARMN | -2.02558 | 10.67927 | 6.51E-14 | 2.62E-12 |
| AP001267.3 | -2.02259 | 5.567766 | 2.36E-46 | 8.21E-43 |
| LINC01354 | -2.01944 | 4.701129 | 1.66E-14 | 7.38E-13 |
| AC015908.2 | -2.01884 | 4.017925 | 2.88E-18 | 2.44E-16 |
| LINC01644 | -2.01863 | 4.693575 | 1.68E-10 | 3.49E-09 |
| LINC01358 | -2.0182 | 3.141436 | 1.09E-13 | 4.24E-12 |
| AC069228.1 | -2.01436 | 3.409241 | 8.61E-06 | 5.39E-05 |
| AC005165.1 | -2.0123 | 6.675342 | 7.41E-11 | 1.64E-09 |
| LINC02269 | -2.01006 | 3.267646 | 4.44E-07 | 3.88E-06 |
| AC104971.3 | -2.00783 | 3.286117 | 4.01E-11 | 9.35E-10 |
| LINC01676 | 2.000501 | 2.498796 | 0.000729 | 0.002493 |
| AC006273.1 | 2.002662 | 4.087514 | 3.86E-09 | 5.83E-08 |
| AC063977.6 | 2.003692 | 3.919501 | 1.32E-05 | 7.79E-05 |
| LINC01979 | 2.00434 | 7.659598 | 2.11E-11 | 5.23E-10 |
| LINC01208 | 2.005964 | 2.482098 | 0.001473 | 0.004561 |
| DLGAP1-AS5 | 2.008144 | 8.362018 | 0.003441 | 0.009253 |
| AP001065.2 | 2.010274 | 3.82027 | 1.87E-05 | 0.000106 |
| LINC01671 | 2.011206 | 6.532052 | 0.001798 | 0.00539 |
| AC148477.3 | 2.011681 | 3.799171 | 0.000541 | 0.001932 |
| TSPEAR-AS2 | 2.012244 | 8.125306 | 1.81E-08 | 2.34E-07 |
| AL354732.1 | 2.012759 | 5.276652 | 1.58E-12 | 4.85E-11 |
| AF240627.1 | 2.014991 | 2.227422 | 0.001485 | 0.004588 |
| AC007336.2 | 2.015722 | 2.37871 | 4.43E-05 | 0.000221 |
| LINC01241 | 2.016116 | 2.182179 | 0.003467 | 0.009311 |
| DIAPH3-AS2 | 2.01896 | 2.123388 | 1.43E-05 | 8.38E-05 |
| AC136475.3 | 2.022248 | 8.985029 | 2.92E-08 | 3.56E-07 |
| VCAN-AS1 | 2.022391 | 3.067348 | 9.66E-07 | 7.84E-06 |
| AL670729.3 | 2.025666 | 2.701692 | 1.04E-07 | 1.08E-06 |
| AC148477.4 | 2.028831 | 4.343329 | 0.000909 | 0.003012 |
| AC243967.2 | 2.029509 | 4.099933 | 2.34E-05 | 0.000127 |
| AP001180.1 | 2.029675 | 2.102885 | 0.001178 | 0.003762 |
| AC024560.1 | 2.030542 | 4.458374 | 2.97E-05 | 0.000157 |
| LINC01910 | 2.034504 | 4.470784 | 1.18E-06 | 9.33E-06 |
| AC108451.2 | 2.034795 | 6.234784 | 0.000441 | 0.00162 |
| AP003027.1 | 2.037652 | 2.141202 | 0.000158 | 0.000667 |
| LINC02294 | 2.040934 | 2.17237 | 0.000982 | 0.003221 |
| LINC01271 | 2.041521 | 4.284199 | 4.17E-10 | 7.98E-09 |
| LINC00501 | 2.041611 | 5.702024 | 2.35E-07 | 2.23E-06 |
| LINC01143 | 2.042908 | 2.595598 | 0.001952 | 0.00578 |
| AC021074.3 | 2.043125 | 2.449173 | 0.002972 | 0.008183 |
| LINC01722 | 2.044264 | 2.878058 | 1.88E-05 | 0.000106 |
| LINC00184 | 2.045468 | 3.855644 | 1.28E-06 | 1.01E-05 |
| LINC02584 | 2.046534 | 3.985681 | 1.36E-06 | 1.07E-05 |
| AC011586.2 | 2.046808 | 2.892017 | 0.000204 | 0.000835 |
| AC112484.3 | 2.04852 | 6.282566 | 2.83E-06 | 2.03E-05 |
| MIR4300HG | 2.049954 | 2.659389 | 0.000267 | 0.001051 |
| MIR5689HG | 2.057224 | 2.774686 | 0.000143 | 0.000611 |
| C2-AS1 | 2.058806 | 3.752218 | 1.92E-08 | 2.46E-07 |
| LINC02519 | 2.059822 | 4.729819 | 2.55E-09 | 4.01E-08 |
| AC138761.3 | 2.063774 | 3.151765 | 0.001004 | 0.003284 |
| LINC02275 | 2.066888 | 2.826922 | 6.20E-06 | 4.04E-05 |
| AC139720.1 | 2.068412 | 4.586299 | 3.07E-06 | 2.17E-05 |
| AFAP1-AS1 | 2.070334 | 12.11277 | 0.000221 | 0.000896 |
| AC106772.1 | 2.071498 | 4.141348 | 2.53E-06 | 1.84E-05 |
| AC026167.1 | 2.071853 | 2.227923 | 0.002934 | 0.008103 |
| LINC02419 | 2.072078 | 2.31988 | 0.000277 | 0.001083 |
| AC004920.1 | 2.076826 | 2.056352 | 0.000694 | 0.002386 |
| Z93403.1 | 2.079567 | 3.781025 | 4.75E-05 | 0.000236 |
| AP003110.1 | 2.079859 | 4.52422 | 0.00055 | 0.001959 |
| LINC01470 | 2.082082 | 3.153365 | 0.002766 | 0.007712 |
| AP000695.1 | 2.08516 | 5.858656 | 9.71E-12 | 2.54E-10 |
| FLJ42969 | 2.088658 | 3.770419 | 4.77E-05 | 0.000237 |
| AC009139.1 | 2.090821 | 2.404802 | 0.00085 | 0.002847 |
| AC135388.1 | 2.091167 | 1.982301 | 0.001534 | 0.004718 |
| LINC00566 | 2.093618 | 2.256788 | 0.000184 | 0.000765 |
| AL355607.2 | 2.094853 | 2.328547 | 0.000119 | 0.000521 |
| AC084346.2 | 2.094883 | 3.072234 | 3.24E-07 | 2.94E-06 |
| AC019117.1 | 2.096503 | 6.747549 | 5.79E-06 | 3.80E-05 |
| AL391832.1 | 2.099067 | 3.259121 | 1.14E-06 | 9.07E-06 |
| LINC02320 | 2.100176 | 3.95202 | 4.01E-06 | 2.74E-05 |
| AL355596.1 | 2.100386 | 3.034284 | 0.000524 | 0.001877 |
| AC079949.2 | 2.100507 | 7.796064 | 1.83E-07 | 1.78E-06 |
| AL078612.2 | 2.101442 | 2.065179 | 3.51E-05 | 0.000182 |
| AC068389.1 | 2.103856 | 2.878849 | 6.98E-07 | 5.86E-06 |
| LINC01553 | 2.103969 | 2.778859 | 7.87E-05 | 0.000363 |
| AL110292.1 | 2.105647 | 2.610567 | 0.001288 | 0.004051 |
| LINC01749 | 2.10636 | 4.346051 | 0.000463 | 0.001687 |
| AC009060.1 | 2.106675 | 2.63069 | 0.000184 | 0.000765 |
| LINC01592 | 2.107831 | 2.928878 | 2.52E-05 | 0.000135 |
| AL022316.1 | 2.109104 | 5.251877 | 3.09E-09 | 4.75E-08 |
| AC034245.1 | 2.110236 | 1.996724 | 0.00086 | 0.002876 |
| AC092167.1 | 2.112232 | 2.322826 | 0.000158 | 0.000667 |
| MAP3K20-AS1 | 2.112383 | 9.66362 | 1.58E-05 | 9.16E-05 |
| LINC02182 | 2.115116 | 3.081365 | 0.00177 | 0.005314 |
| AC099066.2 | 2.115374 | 4.484872 | 4.42E-10 | 8.38E-09 |
| AC008083.1 | 2.117174 | 4.00706 | 5.23E-06 | 3.46E-05 |
| AC244100.3 | 2.12048 | 4.135883 | 3.89E-06 | 2.69E-05 |
| DNAH17-AS1 | 2.125551 | 5.108765 | 1.58E-10 | 3.32E-09 |
| AC012150.1 | 2.125679 | 2.236409 | 9.17E-06 | 5.69E-05 |
| AC069120.1 | 2.128433 | 3.747686 | 0.00098 | 0.003216 |
| AL591468.1 | 2.130349 | 3.715613 | 4.42E-06 | 2.99E-05 |
| LINC02261 | 2.130856 | 3.120009 | 0.000327 | 0.001251 |
| MMP2-AS1 | 2.133606 | 5.022594 | 5.55E-09 | 7.94E-08 |
| AL109935.2 | 2.134202 | 2.000672 | 0.000431 | 0.001588 |
| AC004485.1 | 2.137904 | 1.947505 | 0.001575 | 0.004824 |
| LINC02314 | 2.138257 | 2.05267 | 0.001145 | 0.003672 |
| DLGAP1-AS2 | 2.138862 | 8.941859 | 6.05E-18 | 4.86E-16 |
| C2orf48 | 2.139963 | 6.443582 | 5.59E-11 | 1.27E-09 |
| LINC01526 | 2.142298 | 2.191024 | 0.000225 | 0.000911 |
| AC069277.1 | 2.146483 | 5.032692 | 5.03E-05 | 0.000248 |
| LINC01456 | 2.147644 | 2.943488 | 0.002883 | 0.007984 |
| PANCR | 2.149589 | 2.68813 | 0.002751 | 0.007677 |
| AC004888.1 | 2.150226 | 2.026651 | 3.71E-05 | 0.00019 |
| LINGO1-AS1 | 2.15048 | 1.988299 | 0.000921 | 0.003044 |
| AC087318.1 | 2.150816 | 2.027547 | 0.000354 | 0.00134 |
| LINC01653 | 2.150895 | 2.033985 | 0.001782 | 0.005349 |
| AL359555.1 | 2.152092 | 2.405011 | 9.05E-06 | 5.64E-05 |
| CCAT2 | 2.15555 | 3.559497 | 3.17E-05 | 0.000165 |
| AL158839.1 | 2.157722 | 4.247838 | 2.01E-05 | 0.000112 |
| AC007731.1 | 2.159213 | 2.895139 | 0.002022 | 0.005955 |
| LINC01443 | 2.159379 | 3.965311 | 0.002315 | 0.006674 |
| AL133284.1 | 2.162823 | 4.0363 | 2.73E-05 | 0.000146 |
| AC005534.1 | 2.163871 | 4.14208 | 6.29E-13 | 2.16E-11 |
| AC004817.3 | 2.164417 | 4.098101 | 5.49E-09 | 7.90E-08 |
| AL117382.1 | 2.165008 | 7.662115 | 9.40E-09 | 1.28E-07 |
| AC003965.2 | 2.165139 | 5.724528 | 4.85E-09 | 7.07E-08 |
| AC115837.2 | 2.165149 | 2.316903 | 0.000113 | 0.000499 |
| LINC00410 | 2.165434 | 1.945621 | 0.000694 | 0.002386 |
| AC010894.3 | 2.166099 | 5.673136 | 1.39E-05 | 8.19E-05 |
| AL121832.1 | 2.167265 | 5.177735 | 2.27E-05 | 0.000124 |
| LINC01929 | 2.168035 | 4.487656 | 1.94E-08 | 2.49E-07 |
| AC090192.2 | 2.169149 | 4.751783 | 7.45E-05 | 0.000347 |
| CECR3 | 2.173544 | 3.2312 | 8.10E-05 | 0.000373 |
| LINC02539 | 2.175121 | 2.715313 | 1.65E-05 | 9.50E-05 |
| LINC02111 | 2.175971 | 2.131246 | 0.002859 | 0.007933 |
| PCSK6-AS1 | 2.177095 | 2.925689 | 2.94E-08 | 3.57E-07 |
| AC245884.9 | 2.177599 | 4.700416 | 1.15E-05 | 6.94E-05 |
| AL035045.1 | 2.17809 | 3.021661 | 5.13E-06 | 3.40E-05 |
| AC007663.2 | 2.178231 | 6.147406 | 9.61E-06 | 5.92E-05 |
| AC073283.1 | 2.178958 | 5.309401 | 1.35E-12 | 4.23E-11 |
| AC122108.2 | 2.180829 | 3.839642 | 1.36E-05 | 8.04E-05 |
| AC009021.2 | 2.183586 | 2.133883 | 0.000327 | 0.001251 |
| AC005392.3 | 2.188722 | 2.358872 | 0.00032 | 0.001229 |
| AC027312.1 | 2.189824 | 3.392697 | 0.000323 | 0.001238 |
| Z84468.1 | 2.190334 | 2.401608 | 0.002088 | 0.006112 |
| AC011676.1 | 2.193274 | 3.966539 | 1.63E-12 | 4.96E-11 |
| LINC01391 | 2.193329 | 3.215499 | 6.83E-05 | 0.000322 |
| GAPLINC | 2.193552 | 5.912783 | 3.64E-14 | 1.53E-12 |
| AC008938.1 | 2.19617 | 4.318545 | 7.79E-05 | 0.00036 |
| PROX1-AS1 | 2.198018 | 6.464769 | 2.71E-06 | 1.96E-05 |
| AP000997.2 | 2.198375 | 2.618685 | 0.000862 | 0.002881 |
| AP003717.1 | 2.199338 | 2.633257 | 6.07E-05 | 0.000289 |
| LINC01098 | 2.202097 | 2.796939 | 0.000715 | 0.002451 |
| AC116025.2 | 2.20343 | 4.694379 | 5.63E-11 | 1.27E-09 |
| AC114316.2 | 2.205728 | 4.015321 | 6.40E-05 | 0.000303 |
| CLSTN2-AS1 | 2.208117 | 2.542049 | 0.000308 | 0.001186 |
| AC090159.1 | 2.2083 | 2.478195 | 1.88E-05 | 0.000106 |
| LINC01460 | 2.209724 | 3.995134 | 1.00E-05 | 6.14E-05 |
| ZFPM2-AS1 | 2.212138 | 6.938203 | 1.93E-07 | 1.87E-06 |
| LINC00540 | 2.215836 | 6.63577 | 1.29E-05 | 7.68E-05 |
| LINC02088 | 2.21772 | 2.140121 | 0.001908 | 0.005672 |
| RNF144A-AS1 | 2.222919 | 6.450323 | 6.24E-14 | 2.53E-12 |
| AC083806.3 | 2.225923 | 2.293464 | 3.01E-05 | 0.000159 |
| AL023755.1 | 2.227388 | 4.062989 | 3.40E-06 | 2.39E-05 |
| AC011352.1 | 2.227399 | 3.244833 | 0.000311 | 0.001199 |
| AC022784.2 | 2.227422 | 2.422225 | 0.000268 | 0.001055 |
| LHFPL3-AS1 | 2.227574 | 3.508253 | 0.000156 | 0.00066 |
| AC004264.1 | 2.230919 | 5.995469 | 1.39E-15 | 7.58E-14 |
| FOXD2-AS1 | 2.232869 | 9.235018 | 1.14E-20 | 1.37E-18 |
| AC104365.1 | 2.233492 | 4.0688 | 1.66E-06 | 1.27E-05 |
| AC012668.3 | 2.233674 | 3.285144 | 3.76E-05 | 0.000192 |
| LINC00645 | 2.236265 | 2.705573 | 0.003786 | 0.009998 |
| AC108463.2 | 2.240475 | 3.91432 | 2.76E-16 | 1.72E-14 |
| LINC01440 | 2.241658 | 2.723395 | 0.00128 | 0.004032 |
| AC090398.1 | 2.243939 | 2.023765 | 0.00025 | 0.000997 |
| LINC00603 | 2.244157 | 2.326984 | 0.001724 | 0.005205 |
| AC105219.2 | 2.245937 | 2.048108 | 0.000882 | 0.002937 |
| AL049836.2 | 2.248418 | 2.371564 | 0.000281 | 0.001098 |
| AL512413.1 | 2.249373 | 4.990477 | 4.39E-11 | 1.02E-09 |
| AC011131.1 | 2.25018 | 2.041712 | 0.00073 | 0.002494 |
| AC016687.3 | 2.251032 | 2.321478 | 0.000678 | 0.002341 |
| LINC02030 | 2.251087 | 2.17131 | 0.000633 | 0.002206 |
| AL592494.1 | 2.252738 | 2.19631 | 4.59E-05 | 0.000228 |
| C17orf77 | 2.253463 | 5.488924 | 0.000486 | 0.001759 |
| LINC02571 | 2.256955 | 2.536892 | 0.002345 | 0.006742 |
| AL138916.2 | 2.260037 | 2.058729 | 0.000133 | 0.000575 |
| AC097478.2 | 2.263945 | 3.201983 | 0.001135 | 0.003646 |
| ABCA9-AS1 | 2.268957 | 4.603923 | 1.20E-06 | 9.49E-06 |
| AF015262.1 | 2.269115 | 2.296762 | 4.14E-06 | 2.82E-05 |
| AC022126.1 | 2.270186 | 4.696032 | 6.95E-08 | 7.54E-07 |
| AL589740.1 | 2.270999 | 2.628378 | 0.000101 | 0.000451 |
| HOXB-AS4 | 2.272443 | 6.592104 | 7.58E-08 | 8.14E-07 |
| CASC19 | 2.273281 | 6.647498 | 2.95E-08 | 3.58E-07 |
| AC016746.1 | 2.2733 | 2.086084 | 3.94E-05 | 0.0002 |
| AC095350.1 | 2.275044 | 2.083337 | 0.001565 | 0.004801 |
| AL391152.1 | 2.277285 | 4.16107 | 2.69E-06 | 1.95E-05 |
| AC023866.2 | 2.278169 | 2.379583 | 0.001658 | 0.005036 |
| AC034199.1 | 2.282127 | 4.263626 | 6.82E-12 | 1.84E-10 |
| LINC01748 | 2.284951 | 6.469725 | 1.36E-06 | 1.06E-05 |
| LINC00939 | 2.287623 | 5.965435 | 5.20E-08 | 5.92E-07 |
| AL136418.1 | 2.289777 | 6.410313 | 1.48E-08 | 1.93E-07 |
| AC007277.1 | 2.289786 | 5.75746 | 3.99E-05 | 0.000202 |
| AC017076.1 | 2.291854 | 2.952259 | 5.03E-10 | 9.45E-09 |
| CASC20 | 2.292377 | 4.373497 | 0.000152 | 0.000644 |
| MYOSLID | 2.293167 | 5.852272 | 1.87E-06 | 1.40E-05 |
| AC012467.1 | 2.293675 | 3.875899 | 1.03E-09 | 1.77E-08 |
| LEMD1-AS1 | 2.293976 | 4.2459 | 5.43E-08 | 6.16E-07 |
| LINC01299 | 2.295016 | 2.394204 | 5.96E-05 | 0.000285 |
| AP005233.2 | 2.295633 | 6.175382 | 5.26E-09 | 7.62E-08 |
| LINC01693 | 2.296041 | 2.198332 | 5.85E-05 | 0.000281 |
| LINC01606 | 2.29701 | 4.816373 | 0.000245 | 0.00098 |
| AC016717.2 | 2.298967 | 5.682271 | 0.000103 | 0.000459 |
| LINC02081 | 2.30372 | 5.598569 | 1.40E-09 | 2.34E-08 |
| AC022144.1 | 2.304739 | 7.005071 | 4.77E-13 | 1.67E-11 |
| AL713866.1 | 2.305466 | 2.19626 | 0.002109 | 0.006165 |
| AC012366.1 | 2.305903 | 2.064394 | 0.000709 | 0.002433 |
| CCDC144NL-AS1 | 2.306073 | 8.554779 | 2.40E-08 | 3.00E-07 |
| AL161937.1 | 2.307321 | 2.384547 | 1.91E-05 | 0.000108 |
| AC017002.2 | 2.31169 | 2.484308 | 3.48E-06 | 2.44E-05 |
| AL606537.1 | 2.313284 | 3.200635 | 5.60E-05 | 0.000271 |
| AC004231.1 | 2.314627 | 4.485359 | 0.000132 | 0.000574 |
| LINC01681 | 2.318905 | 2.060798 | 0.001139 | 0.003656 |
| AC006967.3 | 2.321351 | 2.987784 | 0.000168 | 0.000705 |
| AC034229.1 | 2.324029 | 2.425906 | 6.22E-06 | 4.05E-05 |
| CYP4A22-AS1 | 2.325686 | 5.010198 | 1.53E-15 | 8.30E-14 |
| AL157400.2 | 2.333653 | 2.909917 | 5.52E-10 | 1.02E-08 |
| AP003555.2 | 2.335551 | 6.463242 | 1.98E-12 | 5.94E-11 |
| LINCR-0001 | 2.337133 | 6.427485 | 2.60E-09 | 4.07E-08 |
| LINC02108 | 2.346418 | 2.52456 | 1.02E-05 | 6.24E-05 |
| L29074.1 | 2.349495 | 2.608201 | 3.31E-08 | 3.96E-07 |
| LINC01924 | 2.349741 | 2.209483 | 0.000823 | 0.002769 |
| AC107398.3 | 2.352637 | 7.41258 | 0.000193 | 0.000796 |
| AP006748.1 | 2.352725 | 3.217398 | 3.81E-05 | 0.000194 |
| MYO16-AS1 | 2.353005 | 3.811231 | 0.000235 | 0.000947 |
| LINC02345 | 2.354761 | 5.29644 | 2.70E-11 | 6.60E-10 |
| AL445072.1 | 2.35674 | 2.096919 | 0.002043 | 0.006006 |
| AC123905.1 | 2.358596 | 2.398621 | 0.000443 | 0.001629 |
| AL355483.3 | 2.358821 | 2.718585 | 5.49E-07 | 4.70E-06 |
| AL354714.3 | 2.35934 | 2.075218 | 0.000385 | 0.001447 |
| FO680682.1 | 2.363272 | 2.461621 | 4.67E-09 | 6.87E-08 |
| AL391056.1 | 2.364203 | 7.990455 | 1.08E-07 | 1.11E-06 |
| AL356234.2 | 2.364739 | 4.315982 | 1.50E-06 | 1.16E-05 |
| LINC02097 | 2.371642 | 3.118526 | 2.71E-08 | 3.32E-07 |
| AC004704.1 | 2.37282 | 2.506942 | 0.000606 | 0.002132 |
| AC003005.2 | 2.373472 | 2.857074 | 5.91E-08 | 6.61E-07 |
| SLCO4A1-AS1 | 2.377217 | 9.137488 | 7.59E-08 | 8.14E-07 |
| AC012103.1 | 2.377302 | 2.601595 | 0.00317 | 0.008619 |
| AC015849.5 | 2.377436 | 6.575221 | 1.43E-13 | 5.43E-12 |
| LINC01048 | 2.378218 | 2.357664 | 2.41E-05 | 0.00013 |
| AL512604.2 | 2.378677 | 2.631495 | 3.25E-05 | 0.000169 |
| AF121898.1 | 2.3811 | 3.590981 | 0.000327 | 0.001251 |
| AL162151.1 | 2.386266 | 2.02885 | 2.06E-05 | 0.000114 |
| AC009121.1 | 2.387348 | 4.276244 | 1.67E-12 | 5.06E-11 |
| CDKN2A-DT | 2.388442 | 2.346398 | 1.05E-05 | 6.41E-05 |
| AL590483.1 | 2.390084 | 2.915438 | 5.28E-05 | 0.000258 |
| LINC00567 | 2.393442 | 4.168495 | 3.96E-06 | 2.72E-05 |
| LINC02073 | 2.397655 | 4.320962 | 4.99E-10 | 9.38E-09 |
| SLC5A4-AS1 | 2.400374 | 5.303435 | 2.31E-07 | 2.20E-06 |
| AC120042.2 | 2.4014 | 2.413006 | 5.56E-06 | 3.67E-05 |
| FRGCA | 2.403016 | 3.515582 | 0.000112 | 0.000496 |
| AC018943.1 | 2.403244 | 2.05827 | 0.000467 | 0.0017 |
| LINC00523 | 2.410881 | 2.636749 | 0.001879 | 0.005599 |
| AL391422.2 | 2.411198 | 1.964147 | 0.000164 | 0.000688 |
| LINC02405 | 2.41718 | 4.441717 | 1.33E-07 | 1.33E-06 |
| AL356740.3 | 2.419378 | 5.530312 | 3.00E-07 | 2.77E-06 |
| SPATA3-AS1 | 2.419748 | 5.510519 | 1.06E-10 | 2.29E-09 |
| AC006449.1 | 2.424205 | 2.109845 | 1.21E-05 | 7.23E-05 |
| AC008114.1 | 2.425031 | 3.619495 | 1.22E-12 | 3.92E-11 |
| AC122710.3 | 2.427536 | 2.538233 | 6.56E-07 | 5.54E-06 |
| KRT7-AS | 2.427818 | 7.166851 | 1.40E-08 | 1.84E-07 |
| AC026369.2 | 2.431673 | 6.208673 | 6.53E-19 | 6.04E-17 |
| AC104996.1 | 2.434352 | 2.125901 | 6.03E-06 | 3.95E-05 |
| BX322234.2 | 2.448276 | 2.198848 | 0.0003 | 0.001162 |
| LINC01705 | 2.449321 | 5.826536 | 6.10E-06 | 3.99E-05 |
| AC026336.2 | 2.455063 | 2.524014 | 0.000144 | 0.000618 |
| U95743.1 | 2.457723 | 2.151185 | 0.000454 | 0.001661 |
| LINC01819 | 2.458784 | 9.197631 | 0.00018 | 0.00075 |
| AC007950.1 | 2.459151 | 3.711491 | 2.40E-05 | 0.00013 |
| AC083906.3 | 2.469584 | 3.648887 | 5.20E-09 | 7.54E-08 |
| AL137789.1 | 2.471817 | 2.695101 | 6.11E-06 | 3.99E-05 |
| AC011478.1 | 2.472223 | 2.015092 | 0.002574 | 0.007286 |
| H19 | 2.47235 | 13.17215 | 4.90E-06 | 3.27E-05 |
| AC097512.1 | 2.475743 | 2.641341 | 0.001726 | 0.005211 |
| LINC01615 | 2.476162 | 5.304359 | 3.02E-12 | 8.67E-11 |
| LINC01346 | 2.476876 | 2.882207 | 0.000173 | 0.000724 |
| AL109610.1 | 2.477243 | 1.965731 | 0.003252 | 0.008818 |
| DLEU7-AS1 | 2.478034 | 4.783528 | 3.86E-18 | 3.24E-16 |
| AC138625.1 | 2.478244 | 2.32962 | 3.07E-05 | 0.000161 |
| AC022035.1 | 2.478455 | 2.058514 | 3.19E-05 | 0.000167 |
| AC012339.1 | 2.479145 | 2.605334 | 0.003327 | 0.008986 |
| LINC02321 | 2.479651 | 4.444018 | 6.18E-09 | 8.76E-08 |
| AC034154.1 | 2.489289 | 2.221238 | 0.000274 | 0.001074 |
| AC022164.1 | 2.489291 | 2.227524 | 0.001477 | 0.004568 |
| AL020994.1 | 2.489923 | 2.664923 | 0.001742 | 0.005243 |
| AC009988.1 | 2.490308 | 2.756111 | 0.001709 | 0.005167 |
| LINC00114 | 2.49076 | 5.780707 | 0.000103 | 0.000459 |
| GPR1-AS | 2.490939 | 4.541832 | 4.37E-05 | 0.000219 |
| AP005328.1 | 2.498099 | 3.550437 | 0.000193 | 0.000796 |
| AC091808.1 | 2.499481 | 2.176979 | 1.63E-06 | 1.24E-05 |
| AC117465.1 | 2.50025 | 2.745137 | 6.54E-07 | 5.52E-06 |
| HPAT5 | 2.503434 | 2.299926 | 1.60E-05 | 9.23E-05 |
| LINC02267 | 2.509882 | 3.718753 | 0.000791 | 0.002677 |
| AL450267.1 | 2.511103 | 2.431915 | 0.002509 | 0.007134 |
| AC010547.2 | 2.513577 | 5.573791 | 2.22E-05 | 0.000122 |
| AC093763.1 | 2.513595 | 2.038178 | 0.00113 | 0.003634 |
| LINC00626 | 2.513829 | 2.619369 | 1.85E-05 | 0.000105 |
| MIR663AHG | 2.515375 | 5.08388 | 6.79E-08 | 7.43E-07 |
| AF124730.1 | 2.516124 | 3.800508 | 6.57E-07 | 5.54E-06 |
| BX547991.1 | 2.517614 | 3.630958 | 0.00015 | 0.000637 |
| AC120498.4 | 2.522493 | 4.533919 | 1.53E-06 | 1.18E-05 |
| AC090796.1 | 2.522656 | 2.125788 | 8.08E-05 | 0.000372 |
| LINC01744 | 2.524434 | 2.012018 | 0.000866 | 0.002893 |
| AC026310.2 | 2.52484 | 3.375612 | 1.24E-06 | 9.79E-06 |
| DLGAP1-AS3 | 2.528339 | 3.041399 | 5.05E-05 | 0.000249 |
| AC004080.4 | 2.532769 | 2.267261 | 3.24E-05 | 0.000169 |
| AL031665.1 | 2.536755 | 3.983601 | 8.86E-07 | 7.28E-06 |
| LINC02022 | 2.537103 | 2.596352 | 0.002425 | 0.006941 |
| AC006357.1 | 2.540545 | 2.67375 | 0.000673 | 0.002324 |
| AC241644.2 | 2.541946 | 2.258285 | 0.001095 | 0.003541 |
| AC024581.1 | 2.547821 | 2.699976 | 7.43E-05 | 0.000346 |
| LINC01338 | 2.555777 | 2.470042 | 1.45E-06 | 1.12E-05 |
| AC074035.1 | 2.556288 | 2.142347 | 0.000121 | 0.000529 |
| LINC01593 | 2.56089 | 3.411657 | 5.59E-06 | 3.68E-05 |
| AC002076.1 | 2.561257 | 3.212228 | 3.18E-05 | 0.000166 |
| LINC02266 | 2.562487 | 3.682981 | 7.75E-06 | 4.91E-05 |
| AF279873.3 | 2.566646 | 4.601209 | 0.000249 | 0.000992 |
| AL357033.2 | 2.569849 | 4.2339 | 2.59E-06 | 1.88E-05 |
| CASC9 | 2.569983 | 8.728796 | 5.79E-08 | 6.50E-07 |
| FOXC2-AS1 | 2.573033 | 2.43385 | 4.88E-05 | 0.000242 |
| Z94160.1 | 2.578209 | 3.501874 | 2.61E-05 | 0.00014 |
| AC010145.1 | 2.582828 | 2.158316 | 0.003327 | 0.008986 |
| AL445584.2 | 2.583078 | 2.667276 | 0.000433 | 0.001595 |
| AC007529.1 | 2.584708 | 3.21341 | 2.82E-06 | 2.03E-05 |
| AP000842.3 | 2.592504 | 4.419079 | 2.30E-05 | 0.000125 |
| AP000785.1 | 2.594 | 3.0973 | 8.58E-05 | 0.000392 |
| AL354984.1 | 2.597526 | 2.110408 | 0.000626 | 0.002186 |
| AL162574.2 | 2.59768 | 3.277392 | 0.000625 | 0.002183 |
| AC138646.1 | 2.600443 | 2.456069 | 0.00224 | 0.006492 |
| AP001011.1 | 2.604336 | 4.138829 | 5.55E-09 | 7.94E-08 |
| LINC01594 | 2.604704 | 4.581145 | 5.37E-07 | 4.60E-06 |
| AC091806.1 | 2.605069 | 5.723152 | 5.53E-09 | 7.94E-08 |
| AC108676.1 | 2.605963 | 6.42616 | 3.25E-11 | 7.78E-10 |
| AC012506.3 | 2.606472 | 2.34337 | 0.000681 | 0.002346 |
| LINC01254 | 2.60808 | 3.757542 | 0.000408 | 0.001525 |
| AC117386.2 | 2.609526 | 4.753334 | 3.04E-07 | 2.79E-06 |
| AC100782.1 | 2.612552 | 2.857155 | 2.09E-05 | 0.000116 |
| AP001085.1 | 2.61594 | 2.460031 | 7.53E-08 | 8.11E-07 |
| AC108463.3 | 2.617232 | 4.654981 | 4.05E-26 | 1.01E-23 |
| AC025575.2 | 2.618709 | 5.850989 | 7.50E-08 | 8.09E-07 |
| AC068491.3 | 2.61971 | 5.280454 | 3.07E-16 | 1.88E-14 |
| AC011676.2 | 2.620318 | 3.244016 | 1.01E-12 | 3.29E-11 |
| NAALADL2-AS2 | 2.622354 | 3.90313 | 9.89E-06 | 6.06E-05 |
| LINC01446 | 2.623936 | 5.299658 | 0.001301 | 0.004086 |
| AC090774.2 | 2.634967 | 3.837286 | 5.98E-05 | 0.000286 |
| AC144450.1 | 2.635924 | 3.598573 | 7.16E-05 | 0.000335 |
| AC002351.1 | 2.638635 | 2.903914 | 8.33E-05 | 0.000383 |
| LINC02109 | 2.644289 | 4.062448 | 2.27E-06 | 1.68E-05 |
| AC107308.1 | 2.644762 | 3.139286 | 4.00E-06 | 2.74E-05 |
| AC011700.1 | 2.646809 | 4.690357 | 2.82E-06 | 2.03E-05 |
| AC008440.3 | 2.647202 | 5.496643 | 1.91E-09 | 3.10E-08 |
| AC109479.1 | 2.650701 | 3.551562 | 3.82E-09 | 5.78E-08 |
| AL354993.2 | 2.652633 | 5.29854 | 2.79E-08 | 3.41E-07 |
| AC012501.1 | 2.655633 | 2.740725 | 0.000883 | 0.002941 |
| AC004080.2 | 2.658711 | 5.956147 | 2.45E-07 | 2.31E-06 |
| IGF2-AS | 2.659416 | 4.688374 | 1.78E-05 | 0.000101 |
| AC105219.1 | 2.660558 | 4.109288 | 3.99E-11 | 9.32E-10 |
| AL008721.1 | 2.66269 | 2.696748 | 1.59E-06 | 1.22E-05 |
| LINC01694 | 2.663691 | 7.694761 | 9.14E-08 | 9.58E-07 |
| AC124067.4 | 2.665633 | 8.891633 | 9.52E-08 | 9.90E-07 |
| SIX3-AS1 | 2.667502 | 3.417626 | 8.85E-05 | 0.000402 |
| LINC02120 | 2.67621 | 2.257759 | 7.28E-05 | 0.00034 |
| AC010967.1 | 2.678323 | 3.051871 | 6.74E-06 | 4.35E-05 |
| LINC01235 | 2.684655 | 7.521661 | 8.69E-15 | 4.12E-13 |
| AL355483.1 | 2.685416 | 2.193965 | 8.42E-06 | 5.29E-05 |
| PTGES2-AS1 | 2.686036 | 5.756166 | 2.09E-12 | 6.23E-11 |
| LINC01429 | 2.686875 | 3.99274 | 2.34E-10 | 4.69E-09 |
| AC108865.2 | 2.688854 | 3.07111 | 0.000267 | 0.001051 |
| LINC01655 | 2.689716 | 4.121239 | 2.52E-09 | 3.97E-08 |
| AC108174.1 | 2.689819 | 3.340118 | 0.000654 | 0.00227 |
| AC068491.2 | 2.690593 | 2.805979 | 1.52E-09 | 2.54E-08 |
| ELFN1-AS1 | 2.694565 | 8.755328 | 2.29E-09 | 3.62E-08 |
| AC019068.1 | 2.699253 | 2.594944 | 7.88E-05 | 0.000363 |
| AC110772.1 | 2.701255 | 2.492109 | 0.000735 | 0.002509 |
| AL137026.2 | 2.703337 | 2.079026 | 0.000111 | 0.00049 |
| LINC02527 | 2.704049 | 3.030601 | 0.000355 | 0.001342 |
| AL035425.3 | 2.705373 | 7.518071 | 0.002728 | 0.007629 |
| AC003988.1 | 2.707415 | 2.334875 | 0.0023 | 0.006635 |
| AC011298.1 | 2.708658 | 6.449138 | 5.04E-06 | 3.35E-05 |
| AL118511.2 | 2.714816 | 2.847264 | 3.99E-06 | 2.73E-05 |
| AC078860.1 | 2.71719 | 3.505163 | 1.02E-06 | 8.24E-06 |
| AC127894.1 | 2.717616 | 1.919194 | 0.000473 | 0.00172 |
| AL161733.1 | 2.719187 | 2.92882 | 3.04E-05 | 0.00016 |
| AP003351.1 | 2.719768 | 3.064129 | 5.30E-07 | 4.54E-06 |
| ERICH3-AS1 | 2.724399 | 2.485452 | 1.99E-05 | 0.000111 |
| AP001599.1 | 2.725292 | 2.585971 | 0.002303 | 0.006644 |
| LINC01701 | 2.731 | 2.227528 | 0.000261 | 0.001034 |
| PLCH1-AS2 | 2.732072 | 2.316195 | 7.70E-08 | 8.22E-07 |
| ELDR | 2.73275 | 3.344779 | 2.62E-06 | 1.91E-05 |
| AC103993.1 | 2.733255 | 1.933892 | 0.000639 | 0.002225 |
| AP000542.2 | 2.735822 | 2.689361 | 0.000155 | 0.000656 |
| AC022028.2 | 2.736152 | 2.783911 | 6.34E-06 | 4.12E-05 |
| AC123788.1 | 2.739708 | 1.979485 | 0.000263 | 0.00104 |
| AC026782.2 | 2.74104 | 3.24146 | 6.83E-08 | 7.46E-07 |
| LINC02407 | 2.744486 | 4.279145 | 1.13E-15 | 6.35E-14 |
| AC108681.1 | 2.744991 | 2.977013 | 7.12E-09 | 9.98E-08 |
| AC104241.2 | 2.748653 | 2.485252 | 0.000614 | 0.002151 |
| AC025244.1 | 2.759152 | 3.027552 | 0.000498 | 0.001798 |
| AC108865.1 | 2.761468 | 3.737307 | 0.000175 | 0.000729 |
| LINC02196 | 2.763188 | 2.170528 | 0.000138 | 0.000594 |
| AL513324.1 | 2.76748 | 2.04718 | 0.000114 | 0.000502 |
| AC008753.2 | 2.768848 | 2.934384 | 0.001109 | 0.003578 |
| AC005580.1 | 2.771706 | 2.186361 | 0.000192 | 0.000792 |
| LINC02257 | 2.774423 | 4.622712 | 2.59E-08 | 3.20E-07 |
| C15orf54 | 2.780767 | 3.605964 | 1.04E-09 | 1.78E-08 |
| AC015574.1 | 2.780963 | 3.82353 | 0.000255 | 0.001011 |
| AC107057.1 | 2.783022 | 2.049893 | 0.000484 | 0.001752 |
| AL445209.1 | 2.784995 | 2.392559 | 0.001176 | 0.003758 |
| AC109454.3 | 2.786097 | 5.046146 | 0.000212 | 0.000865 |
| AP002992.1 | 2.786983 | 4.685934 | 1.48E-05 | 8.63E-05 |
| AC034223.1 | 2.790083 | 2.403115 | 9.90E-05 | 0.000444 |
| AC044784.1 | 2.799346 | 2.004827 | 0.002195 | 0.006378 |
| AC108482.1 | 2.801324 | 2.331836 | 0.000203 | 0.000834 |
| AC126763.1 | 2.808052 | 3.335426 | 4.13E-07 | 3.64E-06 |
| AC108112.1 | 2.80876 | 3.165281 | 1.88E-07 | 1.82E-06 |
| AL136018.1 | 2.815206 | 3.101413 | 2.26E-05 | 0.000124 |
| AC104462.2 | 2.817999 | 2.088874 | 6.32E-07 | 5.36E-06 |
| LINC02086 | 2.819489 | 9.541373 | 1.89E-09 | 3.08E-08 |
| AC129926.1 | 2.819605 | 3.671375 | 0.000321 | 0.00123 |
| AC104809.1 | 2.820617 | 2.762884 | 4.62E-06 | 3.11E-05 |
| AC099520.1 | 2.824084 | 3.193442 | 3.55E-07 | 3.19E-06 |
| AC016705.1 | 2.843706 | 2.858666 | 1.14E-06 | 9.11E-06 |
| LINC01793 | 2.843841 | 3.407702 | 0.000427 | 0.00158 |
| AC091133.3 | 2.845767 | 2.254541 | 8.72E-05 | 0.000397 |
| AC008514.1 | 2.850197 | 7.019565 | 6.23E-08 | 6.92E-07 |
| AC069061.2 | 2.85515 | 3.421434 | 0.00372 | 0.00987 |
| LINC00524 | 2.856152 | 3.925227 | 1.46E-05 | 8.56E-05 |
| AL008723.1 | 2.858729 | 4.899908 | 3.39E-10 | 6.62E-09 |
| FNDC1-IT1 | 2.860094 | 2.553487 | 1.56E-07 | 1.54E-06 |
| AL353693.1 | 2.875016 | 3.92579 | 4.57E-07 | 3.99E-06 |
| AC008278.2 | 2.879285 | 1.977273 | 8.23E-06 | 5.19E-05 |
| AL035252.2 | 2.879951 | 3.879706 | 4.28E-06 | 2.91E-05 |
| LINC01146 | 2.880064 | 6.104918 | 3.18E-11 | 7.66E-10 |
| AC087636.1 | 2.887231 | 2.002754 | 6.48E-06 | 4.20E-05 |
| AC124067.2 | 2.894821 | 5.813149 | 2.41E-10 | 4.81E-09 |
| AC131532.1 | 2.895841 | 4.508623 | 4.20E-05 | 0.000212 |
| AL049836.1 | 2.89586 | 4.400445 | 1.13E-09 | 1.92E-08 |
| GATA3-AS1 | 2.895974 | 3.138132 | 2.70E-07 | 2.53E-06 |
| AC125603.1 | 2.903791 | 4.045453 | 0.000144 | 0.000617 |
| AL133467.4 | 2.904491 | 4.010639 | 0.001509 | 0.004654 |
| BLACAT1 | 2.906086 | 8.740807 | 1.90E-15 | 1.01E-13 |
| LINC00221 | 2.906674 | 5.331685 | 0.001104 | 0.003566 |
| AC068658.1 | 2.919891 | 4.040939 | 6.05E-05 | 0.000289 |
| LINC02042 | 2.923366 | 3.822173 | 1.19E-07 | 1.21E-06 |
| C8orf31 | 2.934103 | 8.900763 | 1.56E-12 | 4.82E-11 |
| AC025575.1 | 2.938793 | 2.341197 | 4.41E-05 | 0.000221 |
| LINC02466 | 2.941205 | 3.069158 | 0.000425 | 0.001576 |
| AC011294.1 | 2.945383 | 3.914898 | 3.79E-08 | 4.48E-07 |
| AC007750.1 | 2.946461 | 4.810856 | 6.46E-20 | 6.75E-18 |
| AC109588.1 | 2.947112 | 3.113024 | 0.001154 | 0.003696 |
| AC024230.1 | 2.950675 | 3.105736 | 0.000256 | 0.001016 |
| FREM2-AS1 | 2.951853 | 2.083664 | 0.000376 | 0.001414 |
| LINC00534 | 2.952618 | 3.110077 | 1.02E-05 | 6.23E-05 |
| LINC01687 | 2.954929 | 2.873241 | 0.000539 | 0.001927 |
| LINC01549 | 2.956496 | 3.245345 | 0.000676 | 0.002334 |
| AC131097.1 | 2.956552 | 3.639323 | 1.04E-07 | 1.07E-06 |
| AC020656.2 | 2.962975 | 9.299387 | 1.92E-11 | 4.80E-10 |
| AC034228.3 | 2.96422 | 2.158585 | 0.000604 | 0.002126 |
| AC004870.2 | 2.967491 | 4.670693 | 9.65E-06 | 5.94E-05 |
| AP000424.1 | 2.967931 | 4.178826 | 1.55E-07 | 1.53E-06 |
| AL138962.1 | 2.972814 | 2.952128 | 3.29E-07 | 2.98E-06 |
| LINC02029 | 2.975716 | 2.889309 | 1.36E-09 | 2.30E-08 |
| AC090673.1 | 2.976052 | 7.255894 | 3.01E-17 | 2.24E-15 |
| AP000525.1 | 2.985636 | 4.493333 | 8.52E-12 | 2.27E-10 |
| AL359378.1 | 2.98757 | 2.526891 | 3.43E-05 | 0.000178 |
| AC090912.3 | 2.991848 | 4.174304 | 1.84E-10 | 3.81E-09 |
| BOK-AS1 | 2.992241 | 4.423673 | 8.68E-05 | 0.000396 |
| HOTTIP | 2.992684 | 9.166087 | 1.66E-08 | 2.15E-07 |
| LINC02141 | 2.993358 | 3.688326 | 6.10E-06 | 3.99E-05 |
| AC090957.1 | 2.998591 | 2.028715 | 0.000159 | 0.000671 |
| AL589743.4 | 3.007806 | 3.234361 | 5.96E-09 | 8.47E-08 |
| AC012494.1 | 3.007944 | 2.666974 | 3.03E-06 | 2.15E-05 |
| AL121580.1 | 3.008843 | 2.450751 | 1.92E-05 | 0.000108 |
| AC121342.1 | 3.009817 | 2.245277 | 0.00316 | 0.008594 |
| AC087269.1 | 3.011116 | 5.491663 | 3.32E-10 | 6.50E-09 |
| MYB-AS1 | 3.015976 | 3.010859 | 4.23E-10 | 8.08E-09 |
| AC091179.1 | 3.027366 | 5.554731 | 0.000149 | 0.000635 |
| AL138789.1 | 3.030556 | 5.111397 | 1.61E-09 | 2.67E-08 |
| AL133330.2 | 3.035132 | 2.393961 | 0.001547 | 0.004753 |
| AC131097.3 | 3.040112 | 6.381756 | 5.35E-11 | 1.23E-09 |
| LINC02167 | 3.041179 | 2.275652 | 0.001493 | 0.004611 |
| AC004988.1 | 3.048797 | 4.855179 | 1.78E-18 | 1.53E-16 |
| TRPM2-AS | 3.051526 | 8.503316 | 2.65E-13 | 9.70E-12 |
| AC002463.1 | 3.052595 | 2.722324 | 2.30E-05 | 0.000125 |
| AL160408.4 | 3.053271 | 2.52248 | 0.00019 | 0.000784 |
| AC005307.1 | 3.053378 | 3.458167 | 0.000272 | 0.001066 |
| AC005150.1 | 3.061038 | 2.222321 | 0.000253 | 0.001005 |
| AC112493.1 | 3.063665 | 2.837609 | 4.13E-05 | 0.000208 |
| LINC02438 | 3.065871 | 2.68769 | 9.59E-06 | 5.92E-05 |
| LINC02128 | 3.075766 | 2.667909 | 0.000213 | 0.000868 |
| AC074124.1 | 3.082437 | 3.136458 | 2.19E-05 | 0.000121 |
| UCA1 | 3.087358 | 11.13702 | 2.33E-07 | 2.21E-06 |
| AL133467.2 | 3.088424 | 4.626245 | 0.00021 | 0.000858 |
| AC090709.1 | 3.088433 | 4.518924 | 4.85E-06 | 3.24E-05 |
| AC092484.1 | 3.090976 | 2.597268 | 1.51E-05 | 8.77E-05 |
| AC124067.3 | 3.091141 | 3.328395 | 1.70E-07 | 1.67E-06 |
| LINC02331 | 3.095418 | 3.70098 | 3.61E-06 | 2.51E-05 |
| AC134312.5 | 3.097278 | 5.327951 | 4.83E-15 | 2.41E-13 |
| DLX6-AS1 | 3.09827 | 7.21351 | 3.48E-08 | 4.15E-07 |
| LINC02254 | 3.10724 | 4.955617 | 8.24E-07 | 6.81E-06 |
| AC022784.6 | 3.112051 | 3.793196 | 9.99E-11 | 2.16E-09 |
| AC007368.1 | 3.116085 | 3.875771 | 0.000134 | 0.00058 |
| LINC02327 | 3.117 | 4.228967 | 7.17E-05 | 0.000335 |
| AC022075.2 | 3.118586 | 2.645778 | 1.60E-06 | 1.22E-05 |
| AC108752.1 | 3.122584 | 2.286697 | 0.001237 | 0.003916 |
| AC124290.1 | 3.127491 | 2.85244 | 0.000762 | 0.002588 |
| LINC02428 | 3.132497 | 4.697547 | 8.46E-06 | 5.31E-05 |
| LINC00393 | 3.139139 | 4.548063 | 0.000174 | 0.000728 |
| AC000099.1 | 3.143968 | 1.931234 | 9.70E-05 | 0.000437 |
| AL162384.1 | 3.147384 | 3.511894 | 0.000407 | 0.00152 |
| AC119150.1 | 3.149527 | 3.295505 | 3.47E-07 | 3.12E-06 |
| MAGEA10-MAGEA5 | 3.150158 | 2.765607 | 8.44E-05 | 0.000387 |
| AL450468.1 | 3.153818 | 3.039846 | 0.000172 | 0.000719 |
| AC021351.1 | 3.156172 | 3.452064 | 0.000543 | 0.001938 |
| LINC00898 | 3.157428 | 3.3174 | 3.69E-06 | 2.56E-05 |
| AC087273.2 | 3.157921 | 3.870475 | 2.25E-07 | 2.15E-06 |
| AL356417.2 | 3.187356 | 4.405161 | 2.72E-13 | 9.93E-12 |
| AC107958.3 | 3.188296 | 2.36231 | 4.28E-07 | 3.76E-06 |
| AL031674.1 | 3.189028 | 2.497341 | 2.18E-05 | 0.00012 |
| Z98257.1 | 3.205292 | 6.789043 | 2.08E-10 | 4.23E-09 |
| AC026320.1 | 3.211671 | 2.523767 | 0.001008 | 0.003295 |
| LINC01297 | 3.219449 | 3.851309 | 2.79E-06 | 2.01E-05 |
| LINC00460 | 3.219862 | 6.069741 | 9.08E-10 | 1.60E-08 |
| AL138900.2 | 3.229294 | 2.099887 | 5.23E-05 | 0.000256 |
| AL391095.2 | 3.230126 | 2.304624 | 0.000139 | 0.000599 |
| LINC00945 | 3.231818 | 2.694202 | 0.001224 | 0.003883 |
| AC073578.1 | 3.233093 | 2.323777 | 0.000101 | 0.000452 |
| LINC00052 | 3.233583 | 2.376792 | 2.52E-05 | 0.000136 |
| LINC01523 | 3.235959 | 1.962644 | 1.70E-05 | 9.77E-05 |
| AC018558.1 | 3.24474 | 1.969981 | 0.001916 | 0.005688 |
| AL611929.1 | 3.250026 | 2.116638 | 0.000105 | 0.000467 |
| AL035401.1 | 3.251101 | 2.755229 | 0.000372 | 0.001401 |
| AC022784.1 | 3.25363 | 7.975106 | 5.06E-08 | 5.79E-07 |
| AC093627.1 | 3.254276 | 2.451797 | 0.000172 | 0.000719 |
| AC007848.2 | 3.259869 | 3.820777 | 5.57E-05 | 0.00027 |
| LINC01511 | 3.261214 | 4.8311 | 7.77E-06 | 4.92E-05 |
| KIF26B-AS1 | 3.272419 | 5.182263 | 5.19E-17 | 3.61E-15 |
| AC104809.2 | 3.276931 | 6.139486 | 3.33E-07 | 3.01E-06 |
| FIRRE | 3.280549 | 6.566035 | 6.82E-17 | 4.62E-15 |
| LINC01940 | 3.293228 | 4.304127 | 9.77E-05 | 0.000439 |
| AC106799.3 | 3.304346 | 3.614975 | 1.60E-05 | 9.24E-05 |
| LINC01108 | 3.307928 | 4.640673 | 9.27E-07 | 7.57E-06 |
| AC011747.1 | 3.309132 | 3.152653 | 5.07E-07 | 4.38E-06 |
| AL592043.1 | 3.310797 | 3.203803 | 0.000204 | 0.000838 |
| AL080248.1 | 3.312933 | 2.221383 | 0.002872 | 0.007962 |
| AC092112.1 | 3.318264 | 5.93662 | 1.06E-08 | 1.43E-07 |
| LINC01608 | 3.323261 | 3.131803 | 0.001785 | 0.005357 |
| MIR2052HG | 3.323703 | 4.549929 | 7.18E-10 | 1.30E-08 |
| LINC01087 | 3.32405 | 4.647413 | 8.72E-07 | 7.17E-06 |
| AC011900.1 | 3.33197 | 2.228073 | 0.00027 | 0.001062 |
| LINC01981 | 3.33312 | 2.880021 | 2.53E-08 | 3.14E-07 |
| AL157778.1 | 3.336462 | 2.7326 | 0.000114 | 0.000501 |
| PINCR | 3.341357 | 3.344534 | 9.08E-06 | 5.65E-05 |
| AL162413.1 | 3.346128 | 5.536866 | 9.40E-07 | 7.67E-06 |
| AL035530.1 | 3.34938 | 2.194276 | 1.08E-07 | 1.11E-06 |
| AC008443.2 | 3.355877 | 4.434541 | 1.40E-07 | 1.40E-06 |
| AC112178.1 | 3.387043 | 2.83893 | 8.54E-05 | 0.00039 |
| AC093520.1 | 3.387745 | 3.948007 | 2.05E-15 | 1.08E-13 |
| LINC00941 | 3.401883 | 8.085715 | 1.28E-12 | 4.08E-11 |
| AC000061.1 | 3.404821 | 5.021744 | 2.94E-06 | 2.10E-05 |
| AL121721.1 | 3.406561 | 3.824227 | 3.00E-08 | 3.63E-07 |
| LINC02278 | 3.409035 | 2.828684 | 0.000724 | 0.002481 |
| LINC02563 | 3.418465 | 2.997478 | 0.000444 | 0.001632 |
| AC025254.1 | 3.419644 | 2.603851 | 0.000474 | 0.001723 |
| NKX2-1-AS1 | 3.42662 | 3.158579 | 0.001712 | 0.005175 |
| AL391845.2 | 3.42734 | 4.912502 | 7.62E-17 | 5.13E-15 |
| AC009262.1 | 3.434911 | 2.623236 | 3.17E-07 | 2.90E-06 |
| AC019183.1 | 3.43587 | 2.697466 | 1.97E-05 | 0.000111 |
| LINC01667 | 3.437354 | 5.343847 | 5.88E-05 | 0.000283 |
| LINC00648 | 3.437565 | 6.251094 | 5.21E-05 | 0.000256 |
| AC023824.1 | 3.444198 | 3.220083 | 0.000349 | 0.001324 |
| LINC01193 | 3.446816 | 2.766238 | 0.002387 | 0.006847 |
| AC122685.1 | 3.449072 | 2.891612 | 1.39E-05 | 8.21E-05 |
| AC100791.2 | 3.449223 | 3.427137 | 2.09E-08 | 2.65E-07 |
| LINC01162 | 3.4523 | 2.056498 | 0.000311 | 0.001199 |
| AC091489.1 | 3.45434 | 2.4778 | 8.35E-05 | 0.000384 |
| AC093817.1 | 3.458309 | 3.182991 | 1.44E-10 | 3.05E-09 |
| AC016710.1 | 3.460839 | 2.060421 | 0.000685 | 0.002359 |
| AC023469.2 | 3.475471 | 2.075082 | 0.001011 | 0.003302 |
| AC125603.2 | 3.476017 | 5.565026 | 1.04E-05 | 6.35E-05 |
| ZBTB46-AS1 | 3.48394 | 4.574792 | 1.27E-06 | 1.00E-05 |
| AL121904.1 | 3.484002 | 2.230817 | 9.89E-05 | 0.000444 |
| HOXC-AS1 | 3.491029 | 5.600225 | 6.28E-18 | 5.00E-16 |
| AC084128.1 | 3.50929 | 2.606812 | 0.000886 | 0.002946 |
| LINC01632 | 3.511121 | 2.854947 | 0.001574 | 0.004824 |
| AL109615.3 | 3.519903 | 7.983102 | 1.89E-16 | 1.19E-14 |
| DUXAP8 | 3.521227 | 8.905803 | 7.65E-21 | 9.40E-19 |
| LINC01967 | 3.521521 | 3.990245 | 7.63E-08 | 8.16E-07 |
| AC126323.3 | 3.525986 | 2.238115 | 0.000543 | 0.001938 |
| AC092969.1 | 3.539073 | 4.598853 | 2.70E-07 | 2.53E-06 |
| KCCAT198 | 3.54275 | 2.941942 | 4.03E-07 | 3.56E-06 |
| AC010998.1 | 3.545427 | 2.931989 | 3.96E-07 | 3.51E-06 |
| AC106799.1 | 3.554568 | 2.773943 | 9.99E-05 | 0.000448 |
| DLEU1-AS1 | 3.557319 | 3.254971 | 3.68E-15 | 1.89E-13 |
| AC245100.6 | 3.559824 | 5.951831 | 3.20E-07 | 2.92E-06 |
| AC005381.1 | 3.563322 | 5.416775 | 7.01E-07 | 5.88E-06 |
| AP005230.1 | 3.564734 | 4.850085 | 9.01E-13 | 2.94E-11 |
| LINC01925 | 3.568382 | 2.352305 | 0.00082 | 0.002763 |
| AC107419.1 | 3.577127 | 3.862841 | 8.91E-08 | 9.38E-07 |
| AC010486.3 | 3.588083 | 2.106279 | 1.89E-05 | 0.000106 |
| LINC01914 | 3.595183 | 5.021515 | 2.72E-17 | 2.04E-15 |
| AC011632.1 | 3.600438 | 5.722634 | 6.91E-08 | 7.51E-07 |
| AC090673.2 | 3.604296 | 2.299456 | 1.49E-08 | 1.94E-07 |
| AC079160.1 | 3.611263 | 4.653302 | 7.70E-07 | 6.40E-06 |
| AC010099.4 | 3.612313 | 2.613437 | 3.07E-07 | 2.82E-06 |
| AC004080.1 | 3.617766 | 3.339296 | 2.63E-07 | 2.47E-06 |
| AC012499.1 | 3.623216 | 3.243198 | 7.47E-07 | 6.22E-06 |
| AC141930.1 | 3.630311 | 3.115328 | 5.85E-07 | 4.99E-06 |
| AC090502.1 | 3.634412 | 4.734506 | 5.74E-11 | 1.30E-09 |
| HOXA11-AS | 3.635077 | 8.336555 | 2.01E-10 | 4.11E-09 |
| AL929601.1 | 3.635872 | 2.292542 | 0.000284 | 0.001108 |
| AP003900.1 | 3.64306 | 4.005165 | 4.60E-05 | 0.000229 |
| AC068134.2 | 3.646812 | 2.880557 | 1.95E-06 | 1.46E-05 |
| AC016550.2 | 3.648481 | 4.075436 | 9.25E-06 | 5.74E-05 |
| AL355096.1 | 3.653035 | 2.740962 | 1.66E-05 | 9.54E-05 |
| LINC02525 | 3.657277 | 4.525411 | 2.71E-05 | 0.000145 |
| AL445647.1 | 3.66239 | 4.385992 | 7.06E-05 | 0.000331 |
| AC021534.1 | 3.662447 | 2.290659 | 0.000265 | 0.001045 |
| MYCNUT | 3.662786 | 2.422911 | 9.72E-05 | 0.000437 |
| LINC02476 | 3.671884 | 4.295851 | 8.52E-05 | 0.00039 |
| LINC00200 | 3.677723 | 4.640923 | 0.000296 | 0.001148 |
| AC087277.1 | 3.680964 | 3.047347 | 0.000286 | 0.001115 |
| AL121578.3 | 3.682375 | 3.238429 | 2.91E-07 | 2.69E-06 |
| LINC01721 | 3.706631 | 2.155269 | 7.94E-07 | 6.58E-06 |
| AC132807.2 | 3.709476 | 3.875791 | 1.87E-05 | 0.000106 |
| AC010343.3 | 3.712316 | 4.58839 | 2.15E-07 | 2.06E-06 |
| LINC01522 | 3.714757 | 4.970325 | 3.07E-07 | 2.82E-06 |
| AC092198.1 | 3.726556 | 5.544466 | 4.65E-09 | 6.86E-08 |
| LINC01691 | 3.731886 | 2.693048 | 2.50E-10 | 4.97E-09 |
| LINC02424 | 3.752578 | 2.815165 | 0.001153 | 0.003695 |
| LINC01029 | 3.756701 | 3.226873 | 0.002716 | 0.007602 |
| AC091133.2 | 3.765615 | 2.655578 | 3.63E-05 | 0.000187 |
| AC079062.1 | 3.785224 | 8.06836 | 1.22E-05 | 7.31E-05 |
| AC083809.1 | 3.80532 | 6.509382 | 7.90E-08 | 8.40E-07 |
| AL031658.2 | 3.811257 | 3.025392 | 3.33E-05 | 0.000173 |
| LINC02365 | 3.812996 | 5.836033 | 2.14E-10 | 4.33E-09 |
| CU104787.1 | 3.81375 | 2.931659 | 0.000404 | 0.001512 |
| LINC02588 | 3.8149 | 4.758267 | 2.64E-06 | 1.92E-05 |
| AL596442.1 | 3.826799 | 2.760669 | 7.57E-06 | 4.81E-05 |
| LINC01913 | 3.836717 | 5.990582 | 7.71E-09 | 1.07E-07 |
| AC009646.2 | 3.846966 | 3.68302 | 4.14E-05 | 0.000209 |
| AL354863.1 | 3.866213 | 2.751582 | 4.10E-05 | 0.000207 |
| LINC02484 | 3.86987 | 3.394629 | 0.000195 | 0.000802 |
| LINC00601 | 3.878338 | 2.585742 | 4.18E-06 | 2.85E-05 |
| AC096759.2 | 3.881216 | 3.449608 | 3.23E-06 | 2.28E-05 |
| LINC02461 | 3.89916 | 3.056543 | 5.03E-07 | 4.35E-06 |
| C7orf69 | 3.90828 | 3.171301 | 2.20E-14 | 9.50E-13 |
| LINC02532 | 3.920821 | 9.79314 | 9.22E-10 | 1.62E-08 |
| AC007991.2 | 3.925699 | 5.190955 | 4.82E-08 | 5.56E-07 |
| AL161431.1 | 3.92575 | 10.11698 | 6.39E-08 | 7.06E-07 |
| G2E3-AS1 | 3.930729 | 4.898674 | 4.37E-10 | 8.33E-09 |
| LINC02577 | 3.93332 | 7.797017 | 3.22E-14 | 1.36E-12 |
| AC007159.1 | 3.945306 | 3.550813 | 2.29E-06 | 1.69E-05 |
| AC011287.1 | 3.956033 | 5.402146 | 1.35E-09 | 2.28E-08 |
| LINC02293 | 3.95925 | 3.124233 | 1.90E-06 | 1.42E-05 |
| LINC01297 | 3.959759 | 3.542884 | 1.26E-06 | 9.92E-06 |
| AC087857.1 | 3.970209 | 3.492015 | 8.96E-07 | 7.34E-06 |
| LINC01986 | 3.972388 | 2.265158 | 0.001624 | 0.004953 |
| AC055717.2 | 3.978327 | 3.205995 | 6.71E-05 | 0.000317 |
| AL390961.1 | 3.987758 | 3.17134 | 1.15E-05 | 6.92E-05 |
| LINC00326 | 3.989081 | 3.577822 | 6.33E-05 | 0.000301 |
| AC026336.3 | 4.001605 | 6.2428 | 6.75E-05 | 0.000319 |
| AC083967.1 | 4.003073 | 2.547889 | 5.75E-08 | 6.47E-07 |
| HOXC13-AS | 4.011472 | 4.733807 | 7.41E-07 | 6.18E-06 |
| AC093515.1 | 4.021866 | 2.765658 | 3.34E-07 | 3.01E-06 |
| FP325330.3 | 4.025914 | 4.813541 | 8.48E-11 | 1.86E-09 |
| AC122134.1 | 4.040854 | 2.31182 | 2.66E-08 | 3.28E-07 |
| LINC01611 | 4.055976 | 3.771694 | 7.63E-07 | 6.34E-06 |
| AC034223.2 | 4.059826 | 2.628179 | 4.19E-06 | 2.85E-05 |
| LINC01602 | 4.063039 | 5.218359 | 6.87E-06 | 4.41E-05 |
| HOXA10-AS | 4.068952 | 6.567768 | 1.47E-13 | 5.58E-12 |
| AP000943.1 | 4.086279 | 3.9311 | 1.44E-11 | 3.68E-10 |
| LINC02544 | 4.087406 | 4.809222 | 1.60E-12 | 4.89E-11 |
| PURPL | 4.09205 | 6.939033 | 2.10E-10 | 4.27E-09 |
| AC106785.2 | 4.092737 | 2.646024 | 0.000425 | 0.001576 |
| LINC01249 | 4.092908 | 3.5589 | 3.67E-06 | 2.55E-05 |
| LINC01210 | 4.097646 | 4.109162 | 8.87E-08 | 9.36E-07 |
| AC106771.1 | 4.098647 | 3.604094 | 9.07E-05 | 0.000412 |
| AC044839.2 | 4.103289 | 4.305883 | 7.10E-06 | 4.54E-05 |
| AC022639.1 | 4.122872 | 3.563571 | 9.89E-08 | 1.03E-06 |
| LINC00858 | 4.130548 | 6.520306 | 3.80E-14 | 1.59E-12 |
| AC116049.2 | 4.131678 | 3.558766 | 1.43E-07 | 1.43E-06 |
| AC007991.4 | 4.140187 | 5.317905 | 7.05E-11 | 1.57E-09 |
| LINC02418 | 4.151285 | 7.431416 | 3.10E-07 | 2.84E-06 |
| AC061975.6 | 4.153641 | 3.928326 | 5.42E-05 | 0.000264 |
| AC074237.1 | 4.160421 | 2.734263 | 7.35E-06 | 4.69E-05 |
| LINC00454 | 4.182246 | 3.695021 | 1.36E-05 | 8.04E-05 |
| LINC02154 | 4.190223 | 5.238256 | 2.14E-10 | 4.33E-09 |
| AC126323.6 | 4.202678 | 3.077993 | 9.13E-06 | 5.67E-05 |
| ST8SIA6-AS1 | 4.207779 | 7.123308 | 3.65E-07 | 3.27E-06 |
| LINC00922 | 4.213649 | 4.507655 | 1.36E-11 | 3.47E-10 |
| AC090116.1 | 4.219429 | 3.823426 | 8.88E-10 | 1.57E-08 |
| LNCAROD | 4.239255 | 5.259983 | 3.78E-06 | 2.61E-05 |
| AC087612.1 | 4.244399 | 4.267405 | 6.81E-06 | 4.38E-05 |
| AC090283.1 | 4.271205 | 2.868755 | 0.001235 | 0.003912 |
| AC104823.1 | 4.273501 | 4.198742 | 6.37E-08 | 7.05E-07 |
| AC012531.1 | 4.276124 | 6.006961 | 2.90E-20 | 3.26E-18 |
| EVX1-AS | 4.285124 | 3.938296 | 1.84E-08 | 2.37E-07 |
| LINC01854 | 4.324695 | 3.808946 | 8.90E-06 | 5.55E-05 |
| LINC01163 | 4.327113 | 3.963652 | 8.61E-10 | 1.53E-08 |
| AC010789.1 | 4.333935 | 5.190981 | 2.10E-12 | 6.25E-11 |
| AC109830.1 | 4.340102 | 2.473312 | 0.000161 | 0.000677 |
| LINC01807 | 4.346935 | 4.01862 | 4.25E-06 | 2.89E-05 |
| LINC02263 | 4.355262 | 2.475491 | 1.07E-05 | 6.49E-05 |
| FAM230C | 4.369637 | 4.773781 | 0.00032 | 0.001229 |
| AC068580.2 | 4.380526 | 5.278366 | 3.41E-12 | 9.66E-11 |
| AC011287.2 | 4.384412 | 3.371566 | 1.18E-09 | 2.01E-08 |
| AC012363.1 | 4.388426 | 5.276553 | 1.32E-19 | 1.33E-17 |
| MIR548XHG | 4.388474 | 4.903613 | 5.21E-06 | 3.45E-05 |
| LINC01980 | 4.397394 | 6.188783 | 1.25E-06 | 9.86E-06 |
| LINC01711 | 4.409835 | 4.745833 | 3.97E-15 | 2.02E-13 |
| PCAT14 | 4.411335 | 6.421232 | 3.88E-07 | 3.45E-06 |
| AP002478.1 | 4.414114 | 5.090563 | 4.84E-16 | 2.81E-14 |
| AC128707.1 | 4.419575 | 3.041529 | 3.27E-05 | 0.00017 |
| AP002784.1 | 4.42865 | 4.779785 | 2.00E-10 | 4.09E-09 |
| AC073323.1 | 4.447959 | 3.383636 | 1.39E-12 | 4.31E-11 |
| LINC01019 | 4.481625 | 4.043392 | 0.000237 | 0.000952 |
| LINC02241 | 4.492793 | 5.046072 | 3.53E-06 | 2.46E-05 |
| POU6F2-AS2 | 4.497177 | 4.301507 | 5.22E-07 | 4.49E-06 |
| AC019155.3 | 4.497424 | 3.982306 | 2.91E-08 | 3.54E-07 |
| AC074389.2 | 4.497602 | 3.898076 | 2.62E-05 | 0.000141 |
| LINC02492 | 4.521443 | 3.54355 | 1.57E-07 | 1.55E-06 |
| AF127577.3 | 4.54182 | 4.823427 | 2.21E-12 | 6.54E-11 |
| LINC01833 | 4.555185 | 8.359765 | 1.62E-13 | 6.09E-12 |
| FEZF1-AS1 | 4.55557 | 8.989656 | 1.23E-16 | 8.06E-15 |
| AP000943.2 | 4.594656 | 4.741011 | 2.18E-10 | 4.41E-09 |
| LINC02335 | 4.600007 | 3.587108 | 1.80E-05 | 0.000103 |
| AC022031.2 | 4.610094 | 4.096478 | 1.88E-06 | 1.42E-05 |
| AP005057.1 | 4.614666 | 2.954504 | 1.77E-06 | 1.34E-05 |
| AC097478.1 | 4.624464 | 7.10421 | 7.48E-11 | 1.65E-09 |
| HOXC-AS2 | 4.668131 | 6.580845 | 1.19E-32 | 6.88E-30 |
| AC007128.2 | 4.676277 | 5.274437 | 8.92E-19 | 8.09E-17 |
| AL513123.1 | 4.677923 | 4.523616 | 5.56E-13 | 1.92E-11 |
| AC005993.1 | 4.690417 | 4.637731 | 1.57E-10 | 3.30E-09 |
| AC007405.2 | 4.690733 | 3.584388 | 7.88E-12 | 2.11E-10 |
| AL138974.1 | 4.755547 | 2.722203 | 1.05E-05 | 6.37E-05 |
| DSCR4-IT1 | 4.76975 | 2.977751 | 5.62E-06 | 3.70E-05 |
| KCNMB2-AS1 | 4.804671 | 6.864705 | 4.21E-17 | 3.03E-15 |
| LINC02119 | 4.818 | 5.050714 | 2.69E-06 | 1.95E-05 |
| FLJ36000 | 4.818852 | 3.281743 | 4.13E-05 | 0.000208 |
| LHX1-DT | 4.821329 | 4.368002 | 5.64E-09 | 8.05E-08 |
| LINC02434 | 4.824325 | 3.44907 | 5.35E-05 | 0.000261 |
| AC093001.1 | 4.840628 | 7.619998 | 5.96E-08 | 6.65E-07 |
| AL589182.1 | 4.888012 | 2.793108 | 2.69E-08 | 3.30E-07 |
| AL139042.1 | 4.892933 | 3.2116 | 4.11E-05 | 0.000208 |
| AL691420.1 | 4.932176 | 3.079251 | 4.28E-06 | 2.91E-05 |
| LINC01639 | 4.947471 | 3.135332 | 1.02E-06 | 8.24E-06 |
| AC091987.1 | 4.951986 | 3.085772 | 3.06E-07 | 2.81E-06 |
| LINC01614 | 4.952286 | 6.549411 | 4.41E-19 | 4.18E-17 |
| AC093895.1 | 4.956462 | 4.592592 | 4.23E-14 | 1.76E-12 |
| LINC01633 | 4.965667 | 3.357256 | 1.11E-12 | 3.59E-11 |
| LINC01257 | 4.967362 | 4.518187 | 1.85E-06 | 1.39E-05 |
| LINC01742 | 5.027314 | 2.893688 | 3.91E-05 | 0.000199 |
| LINC01287 | 5.11733 | 6.711635 | 3.34E-08 | 3.99E-07 |
| NPSR1-AS1 | 5.130714 | 6.248663 | 1.77E-18 | 1.53E-16 |
| LINC02253 | 5.16051 | 6.843328 | 2.00E-09 | 3.21E-08 |
| AC007128.1 | 5.166966 | 6.406463 | 1.18E-24 | 2.33E-22 |
| AC009055.2 | 5.174278 | 4.58223 | 1.37E-06 | 1.07E-05 |
| LINC00355 | 5.250414 | 5.879033 | 6.14E-10 | 1.13E-08 |
| AC007923.1 | 5.271638 | 3.041072 | 5.12E-08 | 5.86E-07 |
| LINC01518 | 5.28548 | 4.304763 | 4.41E-08 | 5.13E-07 |
| AC113346.1 | 5.351982 | 3.721352 | 1.44E-12 | 4.46E-11 |
| BCAR4 | 5.352399 | 5.981729 | 2.37E-07 | 2.25E-06 |
| LINC01194 | 5.457071 | 5.151328 | 2.77E-07 | 2.58E-06 |
| AC007099.1 | 5.461744 | 5.151074 | 1.88E-10 | 3.88E-09 |
| LINC02526 | 5.473362 | 4.973958 | 3.32E-06 | 2.34E-05 |
| AC106799.2 | 5.478613 | 3.22458 | 2.88E-07 | 2.67E-06 |
| AL356364.1 | 5.486108 | 3.484451 | 3.20E-05 | 0.000167 |
| AP000526.1 | 5.486541 | 3.58653 | 2.91E-15 | 1.52E-13 |
| LINC00973 | 5.494162 | 5.109559 | 1.01E-06 | 8.13E-06 |
| HOTAIR | 5.499054 | 8.047766 | 4.71E-21 | 6.00E-19 |
| AC090809.1 | 5.643919 | 4.780691 | 2.04E-08 | 2.60E-07 |
| ERVMER61-1 | 5.670532 | 4.396636 | 2.61E-07 | 2.46E-06 |
| LINC01419 | 5.691055 | 5.115727 | 2.48E-08 | 3.08E-07 |
| AL139023.1 | 5.697734 | 3.905056 | 2.82E-09 | 4.37E-08 |
| AL117329.1 | 5.704396 | 5.746772 | 4.25E-13 | 1.50E-11 |
| OVAAL | 5.821581 | 5.362906 | 1.92E-06 | 1.44E-05 |
| DSCR4 | 5.836762 | 5.008396 | 7.19E-08 | 7.77E-07 |
| AL139002.1 | 5.861755 | 4.87171 | 1.05E-07 | 1.08E-06 |
| LINC01050 | 5.950819 | 4.15414 | 3.47E-20 | 3.81E-18 |
| AC104794.3 | 6.012905 | 3.619085 | 7.58E-15 | 3.63E-13 |
| AC105460.2 | 6.07361 | 4.600847 | 1.05E-07 | 1.08E-06 |
| LINC02163 | 6.09467 | 5.499685 | 2.91E-22 | 4.05E-20 |
| AC008109.1 | 6.146544 | 4.904211 | 5.16E-10 | 9.64E-09 |
| BANCR | 6.205737 | 7.344837 | 1.71E-09 | 2.81E-08 |
| AC079466.1 | 6.208837 | 7.169991 | 1.20E-08 | 1.60E-07 |
| AC010595.1 | 6.293746 | 4.348877 | 6.92E-10 | 1.26E-08 |
| AC109439.2 | 6.312452 | 3.841379 | 9.63E-07 | 7.82E-06 |
| LINC00392 | 6.409291 | 7.58329 | 2.80E-06 | 2.01E-05 |
| HOXC-AS3 | 6.47704 | 6.339473 | 2.41E-20 | 2.74E-18 |
| AL162582.1 | 6.504918 | 4.004227 | 1.01E-08 | 1.37E-07 |
| LINC01234 | 6.656513 | 8.601907 | 1.07E-17 | 8.42E-16 |
| AC073365.1 | 6.724484 | 5.491576 | 4.10E-09 | 6.12E-08 |
| LINC02474 | 6.741722 | 7.468183 | 5.89E-12 | 1.61E-10 |
| AC106875.1 | 6.750588 | 5.104204 | 3.87E-09 | 5.83E-08 |
| TMEM132D-AS1 | 6.823615 | 7.478106 | 4.39E-08 | 5.11E-07 |
| DSCR8 | 6.885191 | 6.815207 | 2.29E-08 | 2.88E-07 |
| LINC02582 | 6.949288 | 7.903643 | 1.58E-08 | 2.06E-07 |
| AC105460.1 | 7.91213 | 9.915634 | 1.78E-08 | 2.29E-07 |
| MAGEA4-AS1 | 8.037574 | 5.725683 | 5.08E-09 | 7.39E-08 |
| AL499627.1 | 8.342749 | 7.20866 | 5.83E-08 | 6.55E-07 |
